# bis-Dehydroxy-Curcumin Triggers Mitochondrial-Associated Cell Death in Human Colon Cancer Cells through ER-Stress Induced Autophagy

**DOI:** 10.1371/journal.pone.0053664

**Published:** 2013-01-11

**Authors:** Valentina Basile, Silvia Belluti, Erika Ferrari, Chiara Gozzoli, Sonia Ganassi, Daniela Quaglino, Monica Saladini, Carol Imbriano

**Affiliations:** 1 Dipartimento di Scienze della Vita, Università di Modena e Reggio Emilia, via Campi 213/D, Modena, Italy; 2 Dipartimento di Scienze Chimiche e Geologiche, Università di Modena e Reggio Emilia, via Campi 183, Modena, Italy; Institute of Pathology, Germany

## Abstract

**Background:**

The activation of autophagy has been extensively described as a pro-survival strategy, which helps to keep cells alive following deprivation of nutrients/growth factors and other stressful cellular conditions. In addition to cytoprotective effects, autophagy can accompany cell death. Autophagic vacuoles can be observed before or during cell death, but the role of autophagy in the death process is still controversial. A complex interplay between autophagy and apoptosis has come to light, taking into account that numerous genes, such as p53 and Bcl-2 family members, are shared between these two pathways.

**Methodology/Principal Findings:**

In this study we showed a potent and irreversible cytotoxic activity of the stable Curcumin derivative *bis*-DeHydroxyCurcumin (bDHC) on human colon cancer cells, but not on human normal cells. Autophagy is elicited by bDHC before cell death as demonstrated by increased autophagosome formation -measured by electron microscopy, fluorescent LC3 puncta and LC3 lipidation- and autophagic flux -measured by interfering LC3-II turnover. The accumulation of poly-ubiquitinated proteins and ER-stress occurred upstream of autophagy induction and resulted in cell death. Cell cycle and Western blot analyses highlighted the activation of a mitochondrial-dependent apoptosis, which involves caspase 7, 8, 9 and Cytochrome C release. Using pharmacological inhibitions and RNAi experiments, we showed that ER-stress induced autophagy has a major role in triggering bDHC-cell death.

**Conclusion/Significance:**

Our findings describe the mechanism through which bDHC promotes tumor selective inhibition of proliferation, providing unequivocal evidence of the role of autophagy in contrasting the proliferation of colon cancer cells.

## Introduction

Apoptosis, also known as type I cell death, is the best described mechanism of cell death and is morphologically characterized by cell shrinkage, membrane blebbing, nuclear condensation, and formation of apoptotic bodies [1]. An energy-dependent cascade of molecular events coordinates the apoptotic process, which can be distinguished into two main pathways: the extrinsic death receptor pathway and the intrinsic mitochondrial pathway. The two pathways seem to be linked and influenced one another, and both trigger the activation of caspases 3, 6 and 7, proteases targeting hundred proteins and leading to cell demolition [2].

Besides apoptosis, autophagy has been described as an alternative self-destructive cellular process. Autophagy has been long known to provide cellular survival following nutrients/growth factors deprivation or other stressful conditions, and only more recently it has been linked to cell death. Also known as type II cell death, autophagy activation is characterized by the presence of autophagic vacuoles in the cytoplasm, and enlargement of the endoplasmic reticulum (ER) and the Golgi apparatus [3]. Double-membraned autophagic vesicles encapsulate cytoplasm and organelles and, after their fusion with lysosomes, autophagolysosomes degrade their contents [4]. The classic autophagy pathway acts downstream of the mTOR (mammalian target of rapamycin) kinase. When this Ser/Thr kinase is associated in mTORC1 protein complex, it is able to suppress the autophagic machinery. 16 autophagy-related (ATG) proteins have been described to participate to the autophagy pathway [5], and the majority of them play a role in the complex process of double-membraned vesicles formation and growth, downstream of mTORC1 [6]. Although the inhibition of mTORC1 pathway is the best known mechanism through which autophagy is induced, many other signaling cascades and transcriptional events can be involved in the activation of the autophagic process. In particular, various kinases control different steps of this catabolic process, such as AMP-activated protein kinase, Akt, mitogen-activated protein kinase (ERK, p38 and JNK) and protein kinase C [7].

The role of autophagy in cell survival rather than cell death is dependent on cell and tissue context as well as on the nature of the stress stimulus [8].

Apoptotic and autophagic cell death are not mutually exclusive pathways: they can induce cell death simultaneously and cooperatively (for a review see [9]). Autophagic morphologies of cells are observed shortly before or during cell death; although, whether autophagy is the mechanism by which cells actually die and whether cell death is executed by autophagy or with autophagy is still discussed [10].

The distinction between apoptotic and autophagic cell death is even more complicated by two considerations: i) various cellular stresses triggering signal transduction pathways can elicit both apoptosis and autophagy and ii) many proteins essential for autophagy are also involved in apoptotic-cell death, such as ATG5, the transcription factor p53 and the Bcl-2 family members.

p53 is a well known oncosuppressor, activated following a variety of stress stimuli, and responsible for transcriptional regulation of both pro- and anti-apoptotic genes. While the pro-apoptotic genes, such as Bax, Puma and Noxa are up-regulated, the anti-apoptotic ones, as Bcl-2α, are down-regulated by nuclear p53. Also cytoplasmically localized p53 has been shown to be important in controlling the apoptotic response, by inducing Bax oligomerization at the mitochondria or by releasing the pro-apoptotic BH3-only proteins from their anti-apoptotic partners Bcl-2/Bcl-XL [11].

The role of p53 in tumor suppression has been also ascribed to its activity in regulating autophagy. p53-mediated activation of autophagy leads to cell death through transactivation of the autophagy-inducing protein DRAM and inactivation of the mTOR pathway [12], [13]. In opposition, pharmacological inhibition and inactivation of p53 suggest a negative regulation of autophagy through transcription-independent mechanisms [14].

In addition to p53, the Bcl-2 family members are common players of apoptosis and autophagy. They are central to the regulation of the outer mitochondrial membrane permeabilization (MMP), which is responsible for the release into the cytoplasm of proteins mediating cell death, such as Cytochrome C. Bcl-2 proteins have been shown to inhibit autophagy by disrupting the Bcl-2/Bcl-XL-Beclin-1 complexes [15]. It is not clear how Bcl-2 proteins participate to the apoptotic *versus* the autophagic process, but the two different functions could be determined by protein localizations at the mitochondria rather than ER [16]. Relative levels of Bcl-2 and Beclin-1 emerged among the multiple cellular factors controlling whether autophagy contributes to cancer inhibition or survival (reviewed in [17]).

Various natural products and drugs are able to induce cancer cell death through the activation of autophagy or by targeting the pathways of autophagy [18]. Tamoxifen, Imatinib, Resveratrol and Curcumin are examples of molecules exerting their cytotoxic activity towards cancer cells *via* induction of autophagic cell death [17], [18].

Curcumin, the active component found in the rhizome of *Curcuma longa*, has shown therapeutic activity against various tumors. It can inhibit the initiation, progression and tumor cell survival [19]. In particular, mouse models and human clinical trials demonstrated its chemopreventive potential for colorectal cancer [19]. In colorectal carcinoma cell lines, Curcumin inhibits cell proliferation by inducing a G2/M cell cycle arrest or apoptosis when used at high doses [20], [21]. Moreover, the modulation of cellular apoptotic pathways by Curcumin has been recently observed on cancer cells of patients with colorectal cancer [22].

Microarray studies showed that Curcumin-induced apoptosis is regulated by multiple signaling pathways [23], [24]. Curcumin up-regulates the pro-apoptotic proteins (Bax, Bim, Bak, Puma and Noxa) and down-regulates the anti-apoptotic ones (Bcl-2 and Bcl-XL) in different cancer cells, triggering the release of Cytochrome C and the activation of caspase 3 [24]. In human melanoma, HL-60 leukemia and gastric cells, Curcumin activates apoptosis through the Fas receptor/caspase 8 pathway [25], [26], [27]. In addition to the apoptotic activity, Curcumin induces ER stress in various human tumor cells, among which liposarcoma cells [28], non small cell lung cancer cells [29] and leukemia cells [30].

The autophagy process takes part to the anti-proliferative and apoptotic activities of Curcumin, both *in vitro* and *in vivo*: in cancer cells and in a xenograft mouse model, Curcumin and its metabolite Tetrahydrocurcumin inhibit the growth of malignant cells by activating autophagic-cell death *via* Akt/mTOR/p70S6K signaling and ERK1/2 pathways [31], [32], [33]. In glioma initiating cells, Curcumin administration results in tumor suppression because of autophagy-induced differentiation events [34].

**Figure 1:**
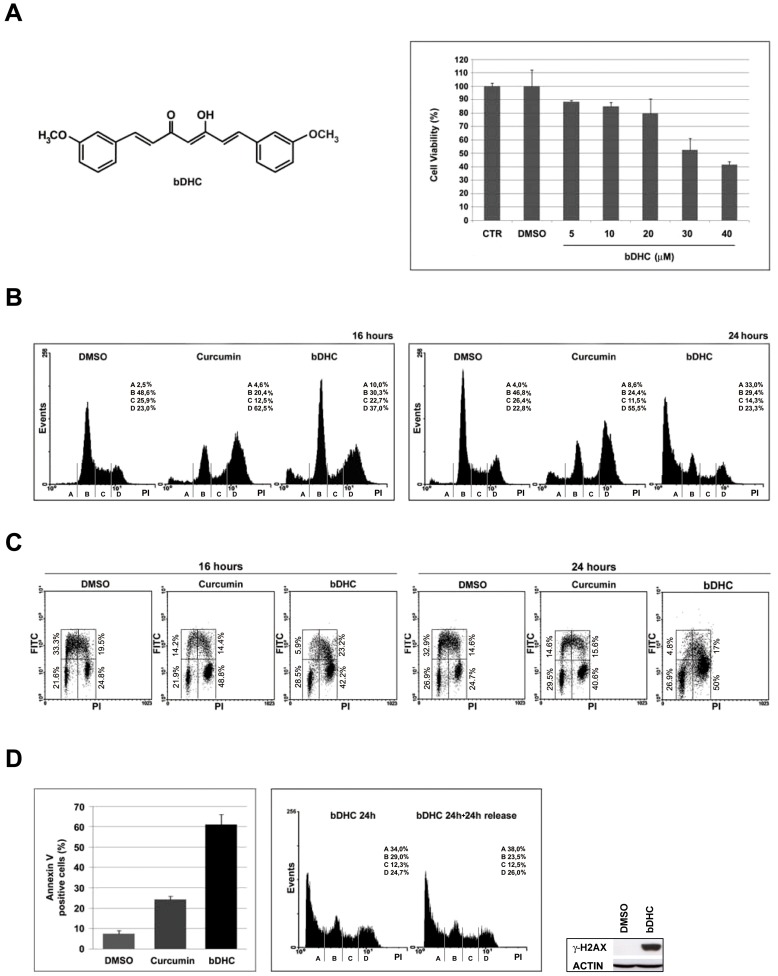
bDHC induces cell cycle impairment and apoptosis in HCT116 cells. **A.** Left panel: Structure of bDHC. Right panel: Dose-response effect of bDHC on cell viability upon 24 hours treatment, compared to control and DMSO-treated cells. **B.** PI/FACS analysis of cell cycle progression after DMSO, Curcumin (10 µM) and bDHC (30 µM) treatments for 16 and 24 hours (left and right panel, respectively). The indicated events are means of ten independent experiments (A = SubG1, B = G0/G1, C = S, D = G2/M). **C.** PI/BrdU bivariate FACS analysis of 16 and 24 hours treatments with DMSO, Curcumin and bDHC. Analysis was gated to exclude SubG1 population. **D.** Left panel: The percentage of Annexin V positive cells upon 24 hours treatment with bDHC is compared to DMSO- and Curcumin-treated cells. Data are means of three independent experiments −/+ SD. Middle panel: PI/monoparametric cell cycle analysis of bDHC-treated cells *versus* bDHC-cells released for 24 hours in fresh medium. Events are indicated as means of three independent experiments (A = SubG1, B = G0/G1, C = S, D = G2/M). Right panel: γ-H2AX expression levels *versus* actin in cells treated with bDHC for 24 hours.

Despite Curcumin inhibition of key molecular pathways of tumorigenesis, clinical trials revealed low bioavailability, limited tissue distribution and rapid metabolism [35]. 90% of Curcumin decomposes rapidly in neutral and basic conditions through oxidation, reduction, glucuronidation and sulfation [28], [29]. To overcome these limitations, natural and synthetic analogs have been synthesized, among which *bis*-DeHydroxyCurcumin (bDHC) (Fig. 1A, left panel). Cells administration of bDHC for 72 h at IC50 concentrations resulted in a slight decrease of anti-proliferative activity compared to Curcumin in androgen-dependent and-independent prostate cancer cell lines and in estrogen-dependent and –independent breast cancer cell lines [36], [37].

In this report, we investigated the tumor-selective inhibitory efficacy of bDHC on the proliferation of human colorectal cancer cells. Compared to Curcumin, bDHC is more active in inducing an irreversible cytotoxic effect in HCT116 and LOVO cells, but not in human normal cells.

The accumulation of poly-ubiquitinated proteins and the induction of ER stress are upstream signals of autophagy, which triggers mitochondrial-dependent apoptosis. Pharmacological and RNAi-mediated inhibition of ER-stress and autophagy highlights that autophagy potentiates the anti-proliferative effect of bDHC. Our studies demonstrate that bDHC acts as a pro-autophagic cytotoxic drug, unraveling its therapeutic potential in fighting selectively tumor development.

## Materials and Methods

### Cell culture and drugs

Human colorectal carcinoma HCT116, HCT116/E6 and HCT116 Bax −/− were generously provided by Bert Vogelstein (Johns Hopkins University School of Medicine, Baltimore, MD). Cells were cultured in Iscove's Modified Dulbecco's Medium (IMDM), supplemented with 10% fetal calf serum (FCS). Primary human fibroblasts (HF) were grown in DMEM 10% FCS, with Glutamine (2 mM) and Gentamicin (55 mg/L). Hepatic fetal human epithelial WRL68 cells and colon adenocarcinoma LOVO cells were maintained in DMEM medium supplemented with 10% FCS. Doubling time has been estimated to be 16 hours for HCT116 and LOVO tumor cells, and 24 hours for HF and WRL68 normal cells.

Curcumin [1,7-bis[3-methoxy-4-hydroxy-phenyl]hepta-1,6-diene-3,5-dione] and bDHC [1,7-bis[3-methoxy-phenyl]hepta-1,6-diene-3,5-dione] were synthesised as previously reported [20]. The purity of synthesized compounds, determined by NMR techniques and combustion analysis, was >98%. Curcumin and bDHC were added to warm medium at 10 µM and 30 µM concentrations, respectively. C3-bDHC was administered to cells at 3 µM concentration. HCT116 cells were incubated with Adriamycin (1.3 µM) for 48 hours. For pharmacological inhibitions, cells were pre-treated for 1 hour and then co-incubated with bDHC for additional 16 or 24 hours with 25 µM ZVAD-fmk (Enzo Life Sciences), 5 µM LEVD-fmk (Enzo Life Sciences), 3 µM Wortmannin (Enzo Life Science), 2 µM Cycloheximide (Sigma Aldrich), 20 µM Chloroquine (Sigma Aldrich), 50 µM Salubrinal (Santa Cruz). Thapsigargin (Sigma Aldrich) was administered to cells for 36 hours at 1 µM concentration.

### Cytometric analysis (FACS)

Flow Cytometric cell cycle analysis was performed as previously described [20]. Indirect fluorescence staining was performed using anti-phospho-Histone H3 (Ser10) (Cell Signaling #9706) and mouse anti-FITC (Dako #F0313) antibodies. Cells were harvested after drug treatments, washed twice with PBS 1×, fixed in 1% formaldehyde for 10 min at 37°C, and post-fixed with 90% methanol o.n. at −20°C. After permeabilization with 0.25% Triton X-100 in PBS 1× for 5 min, cells were stained with primary antibody (1∶25) o.n. at 4°C, and secondary antibody (1∶50) for additional 2 hours at 4°C. Cells were then treated with RNAse A for 40 min, incubated with Propidium Iodide (30 µg/µl) for additional 30 min at 4°C in the dark and analyzed with cytometer.

Apoptotic cells were identified by FACS using Annexin V-FITC conjugate (Bender MedSystems) following the protocol of the manufacturer.

### Cellular uptake studies

bDHC was extracted from cells and culture medium as previously reported [20].

### Crystal Violet assay

The inhibition of proliferation was measured by Crystal Violet staining and the concentration at which cellular growth is inhibited by 50% (IC50) was determined following 24 hours treatment with bDHC. After removal of cell culture medium, the cell monolayer was fixed with methanol and stained with 0.05% Crystal Violet solution in 20% methanol for 30 min. After washes, cells were allowed to dry. The incorporated dye was solubilized in acidic isopropanol (1 N HCl: 2-propanol, 1∶10) and determined spectrophotometrically at 540 nm wavelength. The extracted dye was proportional to cell number. Percentage of cytotoxicity was calculated by comparing the absorbance of treated to untreated cells.

### Acridine Orange staining

HCT116 cells were treated with DMSO and bDHC for 8 and 16 hours. After this, cells were incubated with Acridine Orange (1 µg/ml) for 15 min at 37°C, followed by visualization with a Zeiss AxioSkop 40 fluorescence microscope (Carl Zeiss, Germany).

### Intracellular ATP content

Determination of intracellular ATP content was performed by using ATP bioluminescence assay kit CLSII (Roche), following the manufacturer's protocol.

### Mitochondrial membrane potential

Mitochondrial membrane potential was measured by evaluating the binding of 3,3-Dihexyloxacarbocyanineiodide (DiOC6), a cationic dye that binds to mitochondria with intact membrane potential. Cells were labeled after DMSO or bDHC incubation for 16 and 24 hours with DiOC6 (4 nM) for 40 min at 37°C. Thereafter, cells were washed twice with PBS 1× and the fluorescence intensity was analyzed using Beckman Coulter cytometer.

### Immunoblotting

Cells were lysed in Laemmli sample buffer 1× for total cellular extracts. Nuclear/cytoplasmic extracts were prepared as reported in Ref. [38]. Western blot analysis was performed as previously described [20]. The following primary antibodies were used: anti-p53 DO-1 (Santa Cruz #sc-126), anti-phospo-H3 (Ser10) (Millipore #05-817), anti-H2A acid-patch (Active Motif), anti-p21 (Upstate), anti-actin (Santa-Cruz), anti-PARP1 (Santa Cruz # sc-8007), anti-γH2AX (Millipore, #05-636), anti-GADD153 (F168) (Santa Cruz # sc-575), anti-tubulin (Sigma-Aldrich), anti-cleaved caspases 3, 7, 8, 9 (Cell Signaling), anti-caspase 4 4B9 (Enzo Life Sciences), anti-Bax (N-20) (Santa Cruz #sc-493), anti-Bcl-2 (Santa Cruz #sc-509), anti-Bcl-XL (Santa Cruz #sc-8392), anti-LC3B (Sigma Aldrich #L7543), anti-Ub (Santa Cruz #sc-8017), anti-ATG7 (Sigma Aldrich #A2856), anti-Beclin 1 (Sigma Aldrich #B6061) and anti-H3 (C16) (Santa Cruz #sc-8654). Chemiluminescent detection reagent has been purchased from Millipore (Luminata Classico and Forte Western HRP).

### Isolation of cytosolic fraction by digitonin lysis method

2.000.000 HCT116 cells were washed twice in PBS 1× and then resuspended in digitonin lysis buffer (75 mM NaCl, 1 mM NaH_2_PO_4_, 8 mM Na_2_HPO_4_, 250 mM Sucrose, 190 mg/ml of digitonin, protease inhibitors). Each sample was incubated on ice for 5 minutes and then centrifugated at 15.000×g at 4°C for 30 minutes. The supernatants were collected and used for Western blotting by using anti-Cytochrome C antibody (Santa Cruz # sc-13560).

### Immunofluorescence

Immunofluorescence analysis was performed as previously described [20], [39], using anti-p53 DO-1 (Santa Cruz #sc-126) and anti-LC3B (Sigma Aldrich #L7543) antibodies diluted 1∶100 in PBS+BSA 1%. p53 cellular localization was examined with Zeiss AxioSkop 40 fluorescence microscope (Carl Zeiss, Jena, Germany), images collected with an AxioCam HRc camera and AxioVision version 3.1 software package. Staining of endogenous LC3B was analyzed by confocal microscopy (Leica DM IRE2).

### Plasmids and transient transfection

HCT116 were transiently transfected with Bcl-2, Bcl-XL or carrier plasmids with FuGENE (Promega). Human Bcl-2 and Bcl-XL expression vectors were kindly provided by M. Priault (CNRS IBGC UMR 5095, Bordeaux, France) [40]. Cells were recovered 48 hours after transfection for Western blot and cell cycle analysis.

### Small interfering RNA (siRNA)

HCT116 cells were transfected (Lipofectamine 2000, Invitrogen) with 200 nM of paired ATG7, BCN1 and non-targeting control small interfering RNAs (Sigma Aldrich), as described by Hoyer-Hansen et al. [41]. CHOP siRNA (HSC.RNAI.N004083.10.2 from IDT) was a kind gift of A. Pietrangelo (University of Modena and Reggio Emilia, Modena, Italy). Total extracts and FACS samples were prepared after 72 h upon siRNA transfection.

### RT-PCR analysis

RNA extraction, retrotranscription and semiquantitative PCRs were performed as previously described [42], [43]. Actin and LC3B were amplified with the following oligonucleotides: Actin_For_: 5′-GAGGCCCAGAGCAAGCGT-3′; Actin_Rev_: 5′-GCTCGAAGTCCAGGGCGACG-3′; LC3B_For_:5′-CCTGGAGAAAGAGTGGCATTT-3′; LC3B_Rev_: 5′-GAAGGCAGAAGGGAGTGTGT-3′.

### Scanning electron microscopy (SEM)

Cells grown in monolayer on coverslips were fixed in 1.25% glutaraldehyde in PBS 1× for 30 min R.T. and washed in PBS 1× for three times. After dehydration in graded ethanol solutions, the specimens were critical-point dried with CO_2_ using a Critical Point Dryer 010 Balzer, mounted on aluminium stubs, and sputter-coated with 10 nm gold-palladium in a Coating Unit E 500 (Polaron). Observations were performed by Philips XL-30 scanning electron microscope.

### Transmission Electron Microscopy analysis

Cells, scraped from Petri dishes, were centrifuged at 12000×g for 5′ at 10°C. The resulting pellets were fixed overnight with 2.5% glutaraldehyde (Agar Scientific, Stensted, UK) in Tyrode's buffer, post-fixed for 2 hours in 1% osmium tetroxide (Agar Scientific), dehydrated and embedded in TLV resin (TAAB, Aldermaston, UK). Semithin sections obtained through the whole thickness of pellets were stained with toluidine blue and observed with a Zeiss Axiophot light microscope (Oberkochen, Germany). Ultrathin sections were stained with uranyl acetate and lead citrate and observed with a Jeol 1200 EXII electron microscope (Jeol, Tokyo, Japan).

### Chromatin Immunoprecipitation (ChIP)

ChIPs were performed with chromatin from DMSO and bDHC treated cells for 24 hours as described in Martens *et al.* [44]. PCR oligonucleotides were previously reported [43].

### Statistical analysis

Results are shown as means of at least three independent experiments +/− SD. Statistical analysis was done using one-way ANOVA, followed by LSD test to determine whether there were differences between specific groups.

## Results

### Effects of bDHC on viability and cell cycle progression of HCT116 cells

Full dose-response experiments were performed in HCT116 cells to identify the ability of bDHC to suppress cell growth. bDHC cytotoxicity was assayed through Crystal Violet vital staining method and the 50% inhibitory concentration for cell growth (IC50) was estimated equal to 30 µM, following 24 hours of treatment (Fig. 1A, right panel).

The effect of bDHC on cell cycle progression was investigated by fluorescent-activated cell sorting (FACS) analysis (Fig. 1B). HCT116 cells were treated for 16 and 24 hours with bDHC and the distribution into cell cycle phases was compared to DMSO and Curcumin treated cells. As previously shown [20], Curcumin arrested cells in G2/M phase and halved the population in G0/G1 and S phase within 16 hours; no significant increase of SubG1 events was detected. Differently, bDHC accumulated cells not only in G2/M (from about 23% of control cells to 37% of treated cells) but also in SubG1 phase (from 2.5% to 10%). With prolonged exposure, SubG1 events slightly raised upon Curcumin (from 4% to 8.6%), while an evident increase was detectable with bDHC (from 4% to 33%).

A cell cycle profile was then created by performing PI/BrdU biparametric analysis and using selective gating excluding SubG1 population (Fig. 1C). As expected, Curcumin halved S phase population and doubled G2/M cells. As well as Curcumin, bDHC-treated cells showed a clear decrease of early S population (from about 33% to 5.9% and 4.8% after 16 and 24 hours, respectively) and an accumulation of G2/M events (from about 25% to about 42% and 50%, following 16 and 24 hours), while no significant changes in G0/G1 percentage were detected.

To discriminate between G2 and mitotic populations, the cells were dual probed with PI and a specific antibody against the mitotic marker phospho-histone H3Ser10 using flow cytometry (Fig. S1A). While about 90% of 4n cells were positive to phospho-histone H3Ser10 following Curcumin treatment, only about 2% were detected as mitotic population upon bDHC administration.

The comparison between monoparametric and biparametric analyses (Fig. 1B and Fig. 1C) highlighted that the main effect of bDHC treatment is a G2 block after 16 hours and cell death upon 24 hours.

Cytotoxic activity of bDHC was examined by SEM analysis (Fig. S1B). Deep morphological alterations of the surface, such as loss or enlarged microvilli, membrane “blebs” and apoptotic bodies were present in bDHC treated cells. Moreover, a strong increase of Annexin V positive cells was detected following 24 hours of bDHC administration (61%) compared to DMSO (7%) and Curcumin (24%) treated cells (Fig. 1D, left panel), suggesting the activation of an apoptotic cell death.

To examine the reversibility of bDHC-growth arrest, HCT116 cells treated for 24 hours with bDHC were then washed to remove dead cells and released for 24 hours into fresh medium. An irreversible effect on cell viability was observed, with a clear accumulation of SubG1 events (38%) and no increase in any other phase of the cell cycle (Fig. 1D, middle panel).

Similarly to HCT116, human colon adenocarcinoma LOVO cells showed an evident increase of SubG1 events following bDHC administration for 24 hours (from 2.6% in DMSO to 27.7% in bDHC treated cells) (Fig S1C).

Consistently with the appearance of cells with hypodiploid SubG1 DNA content, bDHC triggered the phosphorylation of the histone variant H2AX (γ-H2AX), whose function is associated with DNA fragmentation during apoptosis [45], both in HCT116 and LOVO cells (Fig. 1D, right panel and Fig. S1C, right panel).

### bDHC induces cell death in HCT116 cells but not in human normal cells

**Figure 2:**
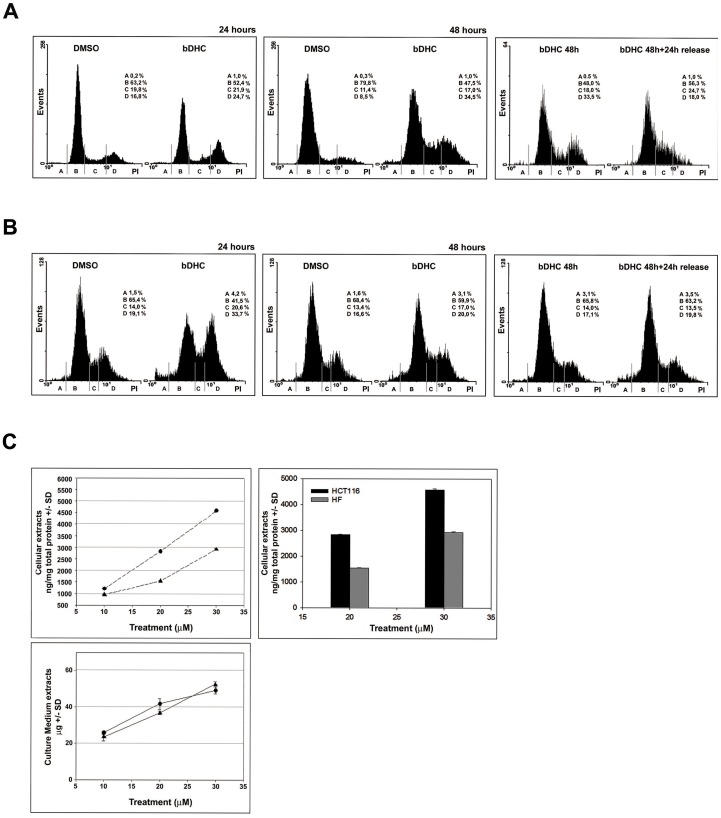
Reversible anti-proliferative activity of bDHC on human normal cells. **A.** PI/FACS analysis of HF cell cycle progression after DMSO and bDHC treatments for 24 and 48 hours (left and middle panel, respectively). PI/monoparametric cell cycle analysis of bDHC-treated cells *versus* bDHC-released cells (right panel) (A = SubG1, B = G0/G1, C = S, D = G2/M). **B.** Left and middle panels: Analysis of cell cycle progression of WRL68 cells following 24 and 48 hours of treatment with DMSO or bDHC. Right panel: PI/FACS monoparametric analysis of WRL68 cells treated with bDHC for 48 hours and then released into fresh medium for additional 24 hours (A = SubG1, B = G0/G1, C = S, D = G2/M). **C.** Quantitative evaluation of bDHC concentration by UV-vis spectroscopy. Upper left panel: bDHC recovered from cell lysates of HCT116 (•) and HF (▴) cells after 24 hours treatment at different concentrations (10, 20 and 30 µM). bDHC recovered from cell pellets (ng) was referred to total cellular proteins (mg), determined using Bradford method. Upper right panel: Comparison of cellular uptake (20 and 30 µM concentrations) in HCT116 (black histograms) and HF cells (grey histograms). Lower panel: bDHC recovered from culture media following incubation of HCT116 (•) and HF (▴) cells with bDHC at 10, 20 and 30 µM concentration. All data were normalized on control (DMSO). Drug amount was determined by reading absorbance at λ_max_ = 393 nm. Reported values are an average of three independent experiments −/+ SD.

We next wondered whether bDHC had a tumor-selective activity. Untransformed human fibroblasts (HF) and hepatic fetal human epithelial normal cells (WRL68), whose doubling time is 24 hours, were treated for 24 and 48 hours with bDHC 30 µM and growth suppressive activity was then investigated by FACS (Fig. 2A and B, left and middle panels). In HF cells, bDHC induced a progressive accumulation of S (from 19.8% to 21.9% and from 11.4% to 17% within 24 and 48 hours, respectively) and G2/M events (from 16.8% to 24.7% within 24 hours, from 8.5% to 34.5% within 48 hours). bDHC administration to WRL68 resulted in an increase of both S and G2/M events as well, but differently from HF cells, maximal effects were detected after 24 hours (from 14% to 20.6% in S phase, and from 19.1% to 33.7% in G2/M phase). Interestingly, neither HF nor WRL68 cells were committed to apoptotic cell death and, coherently, no increase of γ-H2AX expression was observed upon bDHC treatment (Fig. S1D).

To investigate the stability of the cell cycle block induced in normal cells, HF cells were treated with bDHC for 48 hours, then washed and released in drug-free medium for 24 hours. Unlike HCT116, HF cells passed from G2/M to G1 phase (from 48% to 56.3% after the release), and no increase of SubG1 was detected (Fig. 2A, right panel). The reversible effects of bDHC on normal cells were even more evident in WRL68 cells, whose cell cycle distribution after 48 hours and following the release into fresh medium perfectly overlapped that of control cells (Fig. 2B, middle and right panels).

These data hint that bDHC reversibly blocks the cell cycle progression of non-malignant cells without inducing apoptosis.

With the purpose of unraveling the nature of bDHC selectivity towards colon cancer cells rather than normal cells, we performed further studies to estimate the variation of cellular uptake as a function of treatment concentration in HCT116 and HF cell lines. Data were normalized and presented as ng/mg of total proteins (Fig. 2C, upper panels) and culture medium residual amounts (µg) (Fig. 2C, lower panel). Drug uptake increased in a dose-dependent manner in both cell lines, but HCT116 revealed a significant higher uptake as compared to HF cell line at 30 µM : 4575±40 *versus* 2979±21 ng/mg total protein (Fig. 2C, upper right panel).

### bDHC-induced cell death is a caspase-dependent process

**Figure 3:**
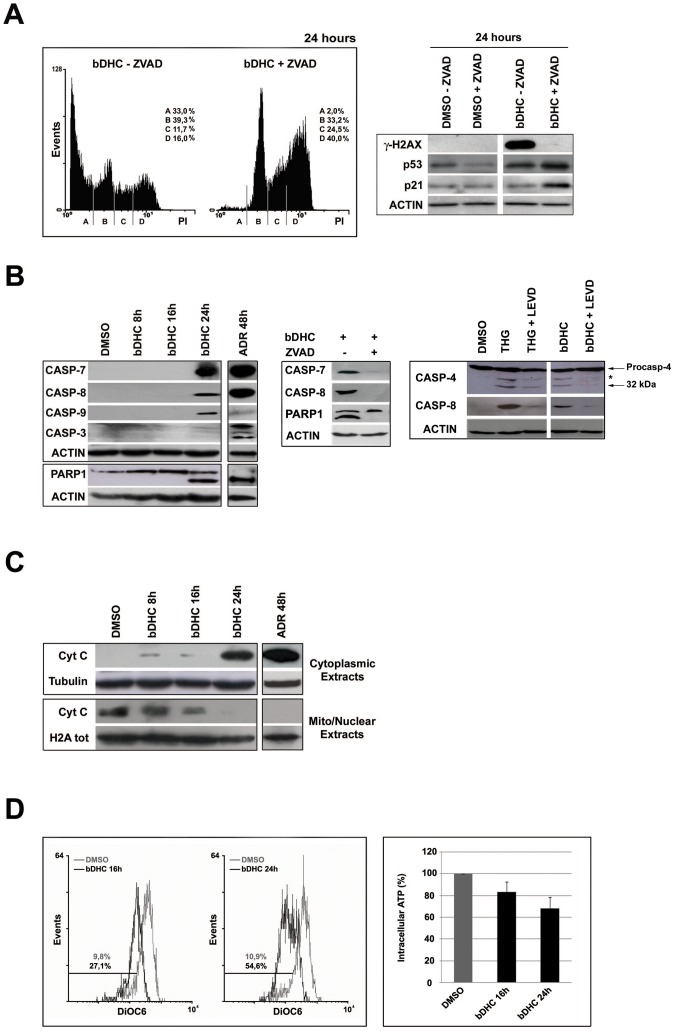
Caspases activation upon bDHC treatment in HCT116 cells. **A.** Left panel: PI/monoparametric analysis of cell cycle progression of bDHC-treated cells with or without ZVAD pre-incubation. The indicated events are means of three independent experiments (A = SubG1, B = G0/G1, C = S, D = G2/M). Right panel: Western blot analysis of the indicated proteins after DMSO and bDHC treatment with or without ZVAD co-incubation. Actin was used as loading control. **B.** Left panel: Expression analysis of cleaved-caspases and cleaved–PARP1 by Western blot following bDHC and Adriamycin administration. Actin was used as loading control. Middle panel: Western blot analysis of cleaved-caspases and PARP1 upon ZVAD pre-treatment compared to bDHC alone. Right panel: Expression levels of cleaved caspase 4 and 8 in HCT116 cells incubated with bDHC for 24 hours and co-treated with LEVD. Thapsigargin (THG) treatment for 36 hours was used as positive control. Protein loading was assessed by probing the blot with anti-actin antibody. The asterisk in caspase 4 blot indicates a band derived from unknown cleavage. **C.** Cytochrome C expression analysis in cytoplasmic and mitochondrial/nuclear extracts from HCT116 treated with bDHC for 8, 16 and 24 hours. Tubulin and total histone H2A were used as loading controls of cytoplasmic and nuclear extracts, respectively. **D.** Left panel: Flow Cytometric analysis of mitochondrial membrane potential (Δψ) by measuring DiOC6 binding in HCT116 cells following administration of bDHC for 16 and 24 hours. The percentage of cells with decreased Δψ is indicated. Right panel: ATP content in HCT116 cells following incubation with bDHC *versus* DMSO (arbitrarily set at 100%).

To explore the contribution of caspases on the execution of apoptosis, we pre-incubated HCT116 cells with the broad-caspase inhibitor ZVAD before treating cells with bDHC for 24 hours (Fig. 3A, left panel). A dramatic drop of SubG1 events was observed concomitantly to a progressive accumulation of cells in S and G2/M phases (from 11.7% to 24.5% in S phase and from 16% to 40% in G2/M, upon ZVAD pre-treatment). The inhibition of apoptosis by ZVAD determined an evident decrease of phosphorylated H2AX (Fig. 3A, right panel and Fig. S2A). The loss of γ-H2AX in ZVAD-bDHC co-treated cells corroborates the hypothesis that bDHC triggers a caspase-dependent cell death, as γ-H2AX formation has been shown to be an early chromatin modification downstream from caspase activation during apoptosis [45].

Interestingly, apoptosis suppression increased the expression levels of both p53 and p21, key regulators of the cell cycle (Fig. 3A, right panel and Fig. S2A).

The activation of individual caspases was then investigated by Western blot upon 8, 16 and 24 hours of treatment (Fig. 3B, left panel). Caspases 7, 8, 9 but not the executioner caspase 3, were clearly cleaved by 24 hours bDHC-incubation. The treatment with the anti-tumor drug Adriamycin demonstrated a fully functional caspase system, which includes caspase 3, in HCT116 cells.

We then explored the impact of caspases activation on proteolysis of poly (ADP-ribose) polymerase 1 (PARP1) substrate (Fig. 3B, left panel). Although caspase 3 was not detected at 24 hours, the 89 KDa fragment of PARP1 was observed, suggesting a redundancy between the executioner caspases. Pre-treatment of bDHC-cells with ZVAD completely abolished the cleavage of pro-caspases and PARP-1, consistently with apoptosis suppression (Fig. 3B, middle panel).

A major caspase activation pathway is the Cytochrome C-initiated pathway, which is triggered by the permeabilization of the mitochondrial outer membrane. Cellular fractionation followed by Western blot showed Cytochrome C release into the cytoplasm upon 24 hours of bDHC treatment (Fig. 3C and Fig. S2B).

Changes in the mitochondrial potential of bDHC-treated cells have been further investigated by labeling cells with DiOC6, a strong cationic dye that binds to undamaged mitochondria with intact membrane potential [46]. A clear decrease in the binding of DiOC6 was observed in cells treated with bDHC for 16 and 24 hours with respect to control cells, indicating the loss of mitochondrial transmembrane potential (Δψ) (Fig. 3D, left panel).

Finally, a time-dependent decrease of intracellular ATP levels was detected (Fig. 3D, right panel), hinting at a compromised bioenergetic function of mitochondria triggered by mitochondrial inner membrane permeabilization with Δψ loss [47].

### Role of the Bcl-2 family members in bDHC-induced apoptosis

The intrinsic pathway of apoptosis is controlled by Bcl-2 family members, which regulate mitochondrial outer membrane integrity.

**Figure 4:**
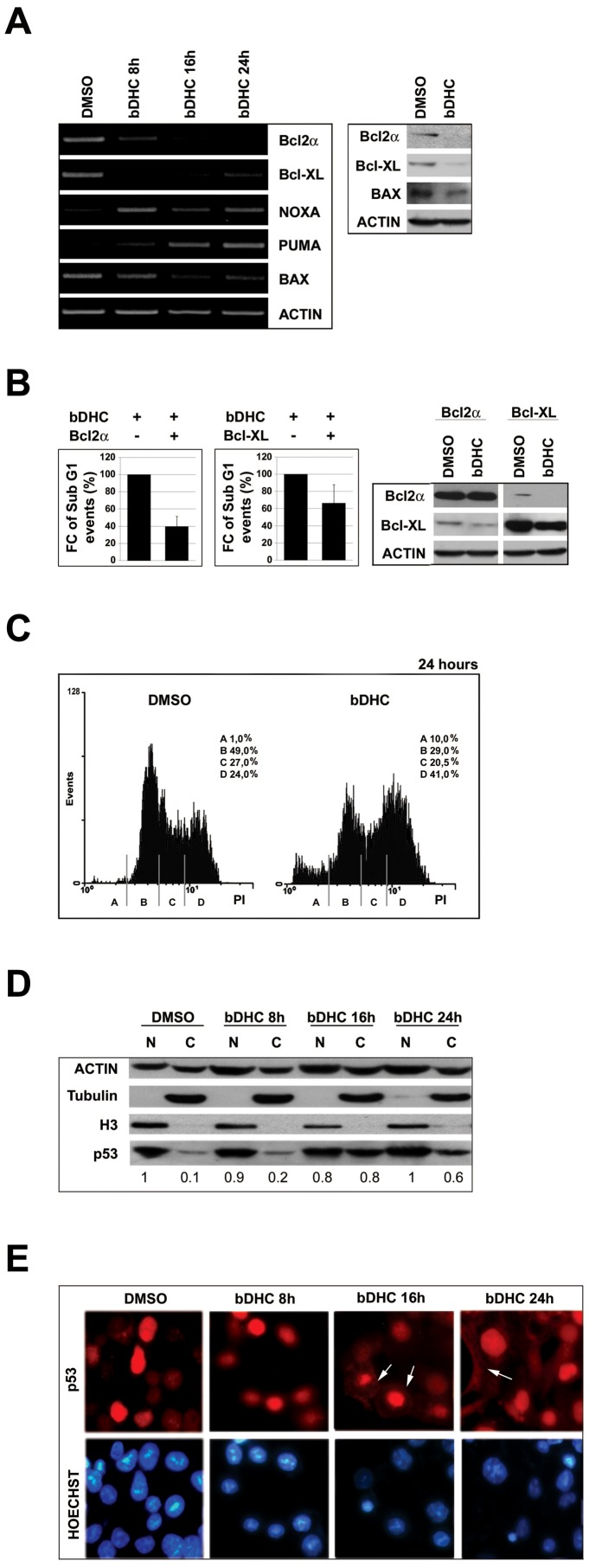
Role of Bcl-2 family members in bDHC-induced apoptosis. **A.** Left panel: RT-PCR analysis of the indicated Bcl-2 family genes upon bDHC treatment for different times *versus* DMSO. Right panel: Protein expression levels of Bcl-2α, Bcl-XL and Bax following 24 hours treatment with bDHC. **B.** SubG1 (left panel) and Western blot analysis (right panel) of HCT116 cells upon transient transfection of Bcl-2α or Bcl-XL and treatment with DMSO or bDHC. Data are reported as fold change (FC) of SubG1 population in transfected cells treated with bDHC relative to bDHC-cells (arbitrarily set at 100%). **C.** PI/FACS analysis of cell cycle progression of HCT116 Bax −/− cells after DMSO and bDHC incubation for 24 hours. **D.** Western blot analysis of p53 expression in nuclear and cytosolic extracts of cells treated with DMSO and bDHC for 8, 16 and 24 hours. Tubulin and histone H3 were used as loading controls of cytosolic and nuclear extracts. The intensity of immunoreactive bands was quantitated to actin (basal nuclear p53 arbitrarily set at 1). Values are means of three independent experiments. **E.** Immunofluorescence analysis of endogenous p53 cellular localization in HCT116 following time-dependent exposure to bDHC. p53 cytoplasmic localization is indicated by white arrows.

By RT-PCRs we investigated mRNA levels of the Bcl-2 anti-apoptotic genes: while Bcl-2α decreased in a time-dependent manner, Bcl-XL was mainly reduced upon 8 hours of treatment (Fig. 4A, left panel). A clear decrease of Bcl-2α and Bcl-XL protein levels was detected upon 24 hours treatment (Fig. 4A, right panel and Fig. S2C).

Given the significance of Bcl-2α and Bcl-XL as suppressors of apoptosis, single gene transfections were performed to determine whether either protein can inhibit bDHC-induced cell death. SubG1 events detected upon bDHC treatment were reduced of about 60% in Bcl-2α and 40% in Bcl-XL overexpressing cells, indicating a more important role for Bcl-2α rather than Bcl-XL in bDHC-apoptotic cell death (Fig. 4B).

As to pro-apoptotic genes, while transcription levels of the BH3-only Noxa and Puma raised compared to control cells, no increase was observed for Bax mRNA and protein levels (Fig. 4A, left and right panels). To shed light on the role of Bax in mediating bDHC-cell death process, we switched to HCT116 Bax −/− cells (Fig. 4C). Compared to HCT116, bDHC-induced apoptosis was partially suppressed (from 1% in control cells to only 10% in treated-cells), in behalf of an increase of G2/M phase population (from 24% to 41%), thus indicating that Bax contributes to bDHC induced cell death.

Despite the participation of Bax to the apoptotic process, the lack of its mRNA up-regulation suggests the activation of a p53 transcriptional-independent apoptotic pathway. In fact cytosolic/mitochondrial p53 could activate Bax/Bak without direct regulation of gene expression [48]. As it concerns Puma and Noxa, they have been shown to be activated through p53-dependent and -independent mechanisms. Puma can be induced by ER stress [49], and regulated by transcription factors other than p53, including FOXO3a, p73 and E2F [50]–[52]. Noxa mRNA can be induced by the proteasomal inhibitor MG132, in p53 null human cell lines [53].

To investigate whether p53 could play a role in mitochondrial-dependent apoptosis rather than in the transcriptional control of the apoptotic pathway, we analyzed the expression and cellular localization of endogenous p53 following bDHC administration. Western blot highlighted an increase of p53 levels into cytosolic fractions of cells treated for 16 and 24 hours with bDHC (Fig. 4D). Similarly, cytoplasmic p53 was detected by Immunofluorescence only following 16 and 24 hours of bDHC incubation (Fig. 4E, arrows). The accumulation of p53 protein outside the nuclear compartment suggests an important role of p53 mainly into cytoplasmic molecular mechanisms.

### Activation of the endoplasmic reticulum stress response following bDHC treatment

**Figure 5:**
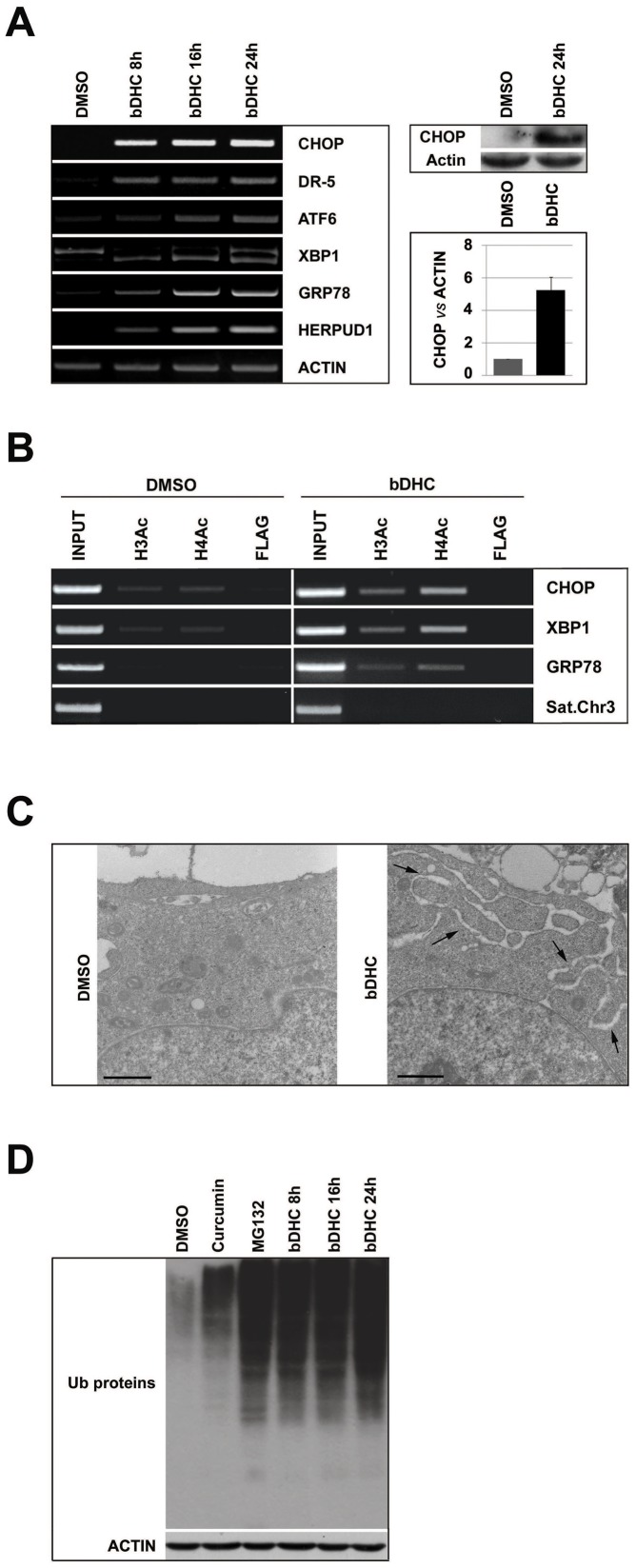
Activation of ER stress response in bDHC-treated HCT116 cells. **A.** Left panel: time-course mRNA expression analysis by RT-PCR of the indicated genes. CHOP protein expression (right upper panel) and quantification (right lower panel) after 24 hours treatment with bDHC *versus* DMSO. **B.** Changes in histones acetylation upon bDHC treatment. Anti-Acetylated histone H3 and H4 have been used for ChIP analysis of the indicated regulatory regions. **C.** Transmission Electron Microscopy analysis of ER in control and bDHC-treated cells. The black arrows indicate the expanded ER. Scale bar: 1 µm. **D.** Increase of protein ubiquitination in Curcumin (24 hours), MG132 (24 hours) and bDHC lysates (8, 16, 24 hours) by Western blot analysis. Actin was used as loading control.

As well as described for the pro-apoptotic genes, also anti-apoptotic Bcl-2 family members can be transcriptionally regulated in a p53-independent way. The transcriptional down-regulation of Bcl-2α following bDHC treatment could be ascribed to different transcriptional regulators, among which CHOP (C/EBP homologous protein). Indeed, an increase of CHOP expression levels was observed by RT-PCR and Western blot in bDHC-treated cells (Fig. 5A). CHOP is a transcription factor induced under ER stress, which triggers an ER-specific cascade for implementation of apoptosis [54]. mRNA levels of several ER-stress induced genes were studied (Fig. 5A, left panel). We observed a transcriptional increase of the pro-apoptotic CHOP target gene DR-5 (death receptor-5), and of two main CHOP transcriptional activators, ATF6 and the ER-induced spliced variant of XBP1. Other ER-stress markers, such as GRP78, a chaperone that binds to unfolded proteins, and HERPUD1 were up-regulated in a time-dependent manner.

A considerable increase of H3 and H4 acetylation was observed by ChIP in regulatory regions of ER-stress genes, consistently with their transcriptional activation (Fig. 5B).

The analysis of cells treated for 16 hours with bDHC by electron microscope revealed enlargement and dilation of the ER, corroborating the activation of ER stress (Fig. 5C).

Severely damaged ER functions have been shown to induce apoptosis. In addition to CHOP, processing of caspases 12, 4, 3, 6, 7, 8, and 9 has been observed to play a role in ER stress-induced apoptosis. In particular, caspase 12 in rodents and caspase 4 in humans can initiate a specific cascade independent of mitochondria, linking ER stress to apoptosis [55], [56], [57]. We therefore examined caspase 4 activation following bDHC administration, compared to Thapsigargin (THG), which is able to trigger ER stress-apoptosis in HCT116 cells [58]. Although to a lesser degree with respect to THG, cleavage of caspase 4 (32 kDa subunit) was induced by bDHC and reverted by co-incubation with the caspase 4 inhibitor LEVD (Fig. 3C, right panel). Interestingly, caspase 4 inhibition resulted in a clear decrease of caspase 8 processing, suggesting that caspase 4 acts upstream of caspase 8.

Curcumin-induced proteasomal dysfunction and inhibition were shown to contribute to Curcumin-ER stress activation [59], [60]: we therefore examined proteasomal function by Western blot analysis of bDHC-total cellular extracts with anti-poly-ubiquitin antibody. Figure 5D highlights an accumulation of poly-ubiquitinated proteins upon Curcumin administration for 24 hours. Exposure to bDHC, even after 8 hours, resulted in a more robust increase of poly-ubiquitinated proteins than Curcumin, comparable to the effect of the well known proteasome inhibitor MG132.

These data suggest that the proteasome can't degrade the ubiquitinated proteins accumulated in the ER lumen and that the process of ER stress is already maximally stimulated by bDHC upon 8 hours.

### bDHC induces autophagy in p53-positive cells

**Figure 6:**
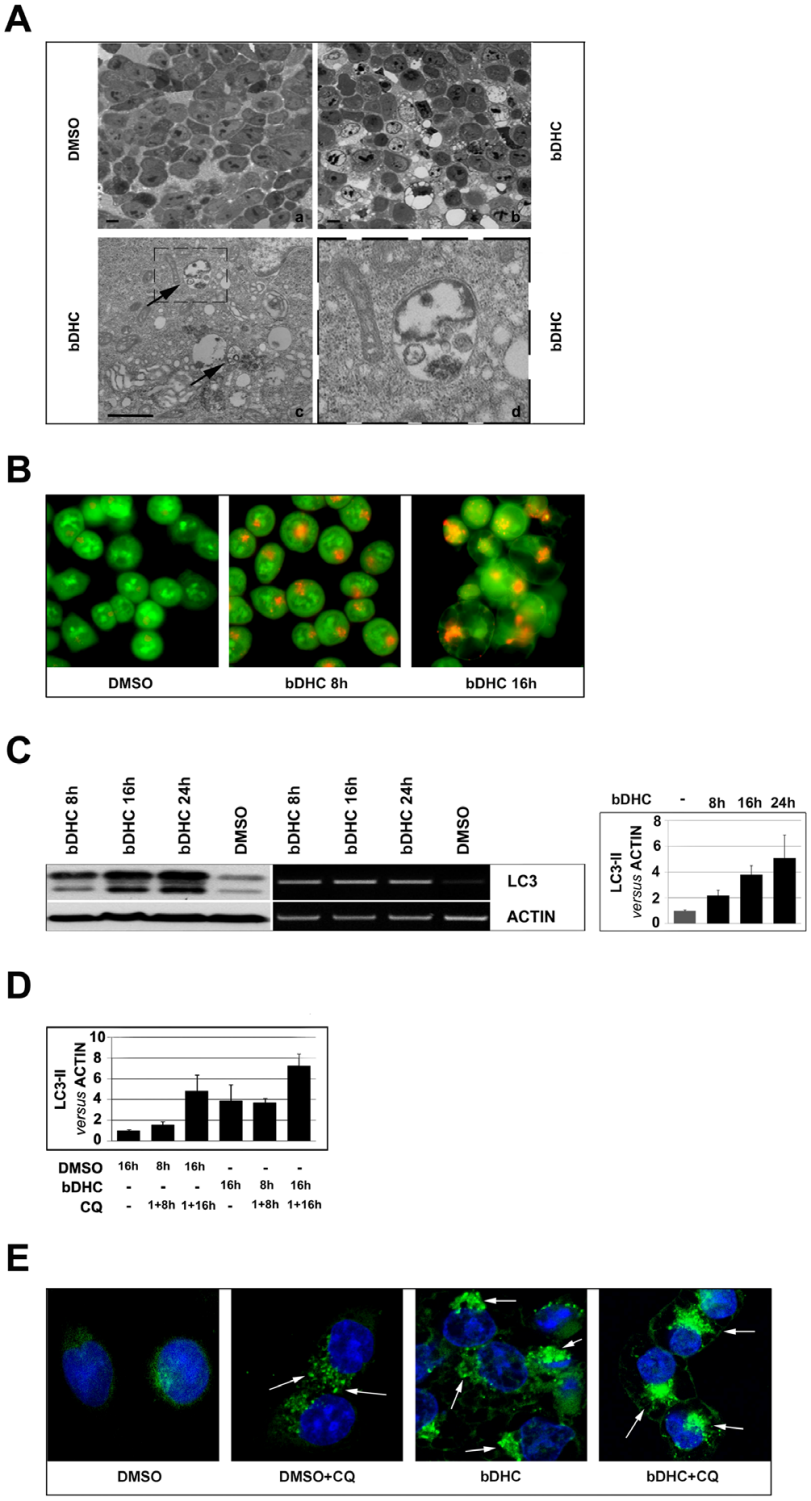
bDHC induces autophagy in HCT116 cells. **A.** Optical (*a* and *b* photomicrographs) and Scanning Electron Microscopy (*c* and *d* photomicrographs) representative images of cells treated with DMSO and bDHC for 24 hours. Photomicrograph of panel *c* shows the presence of autophagolysosomes in bDHC treated cells. Enlargement of the dashed box (panel *d*) illustrates a double-membraned autophagosome. Scale bar *a* and *b* panels: 10 µm; *c* panel: 1 µm. **B.** Detection of bDHC-induced autophagosomes formation by fluorescence microscopy following staining with Acridine Orange. **C.** Left panel: Western blot and RT-PCR analysis of LC3 expression at 8, 16 and 24 hours of bDHC exposure in HCT116 cells. Right panel shows the levels of LC3-II *versus* actin following time-dependent incubation with bDHC. Values are mean of six independent experiments −/+ SD. **D.** Quantification of LC3-II detected by Western blot analysis following pre-incubation (1 h) of DMSO and bDHC with Chloroquine (CQ). The levels of LC3-II have been normalized to actin. **E.** Fluorescence staining of endogenous LC3 following incubation of HCT116 cells with bDHC for 24 hours, with or without Chloroquine (CQ). LC3-stained autophagic compartments are indicated by white arrows.

Phase-contrast microscopy analysis of bDHC-treated cells highlighted the presence of cytoplasmic vacuoles (data not shown). We performed ultrastructural analysis by using electron microscopy to further investigate the morphological changes induced by bDHC upon 16 hours of incubation. Compared to control cells, bDHC treated cells showed double membrane vacuolar structures with the morphological features of autophagosomes (Fig. 6A, arrows).

Formation of autophagolysosomes was also detected by fluorescence microscopy following staining with the lysosomotropic agent Acridine Orange, whose protonated red fluorescent form accumulates in acidic compartments [61]. While control cells showed green fluorescence with minimal cytoplasmic red components, corresponding to lysosomes, bDHC-treated cells displayed considerable red fluorescence, caused by the formation and accumulation of autophagolysosomes (Fig. 6B).

To validate the hypothesis that bDHC could induce autophagy, we monitored by Western blot the formation of LC3-II, which is regarded as autophagy marker [62]. bDHC induced a time-dependent expression of the microtubule-associated protein light chain 3 (LC3-I), and the accumulation of its processed form (LC3-II) (Fig. 6C). Quantification of LC3-II versus actin levels highlighted an increase of about 5 fold of LC3-II in cells treated for 24 hours with bDHC, compared to DMSO (Fig. 6C, right panel). Figure S3A shows the quantification of the conversion of LC3-I into the phosphatidylethanolamine conjugate LC3-II (LC3-II/LC3-I ratio).

Interestingly, bDHC was not able to increase LC3-II in HF cells after 24 and 48 hours (Fig. S4A), suggesting that bDHC-cell death could be related to autophagy activation.

The increase of LC3-II and the appearance of autophagosomes are not measure of the autophagic flux, but can reflect the inhibition of autophagosome clearance [63]. To verify whether autophagic flux was occurring in bDHC-treated cells, we prevented lysosomal degradation by using Chloroquine, that neutralizes the lysosomal pH [64]. As shown by the quantification of LC3-II levels with respect to actin, the amount of LC3-II induced by bDHC strongly increased in the presence of the inhibitor, corroborating a time dependent activation of autophagy (Fig. 6D).

Finally, we monitored bDHC-induced autophagy through Immunofluorescence, measured as an increase in punctuate endogenous LC3. Figure 6E shows the changes in LC3 localization upon incubation with bDHC, compared to HCT116 control cells. LC3-stained autophagic compartments are indicated by arrows. As already observed (Fig. 6D), Chloroquine induced an increase in the level of LC3 puncta in control cells and had an additive effect when administered with bDHC.

p53 has been described to regulate the activation of autophagy: nuclear p53 can participate to autophagy induction, while cytoplasmic p53 inhibits autophagy through a transcriptional-independent mechanism. We therefore analyzed the effects of bDHC administration to HCT116/E6 cells, in which p53 is degraded by the viral E6 protein. Under normal conditions HCT116/E6 showed higher levels of LC3-II compared to HCT116 wt, but no increase was observed following bDHC treatment (Fig. S4B, upper panels). Moreover, HCT116/E6 cells were less susceptible than wt cells to bDHC-induced apoptosis, as indicated by the lower percentage of SubG1 events (from 33% in bDHC-treated HCT116 to 16% in bDHC-HCT116/E6 cells) (Fig. S4B, lower left panel). The high levels of poly-ubiquitinated proteins observed in HCT116/E6 cells suggest that bDHC can induce ER stress also in p53 deficient cells, but it doesn't elicit the activation of autophagy, which can contribute to drug-induced cell death (Fig. S4B, lower right panel).

### Autophagy activation triggered by bDHC potentiates its pro-death activity

To investigate whether autophagy and apoptosis participate independently in parallel pathways or they affect one another, we co-treated cells with bDHC and inhibitors of autophagy or apoptosis.

**Figure 7:**
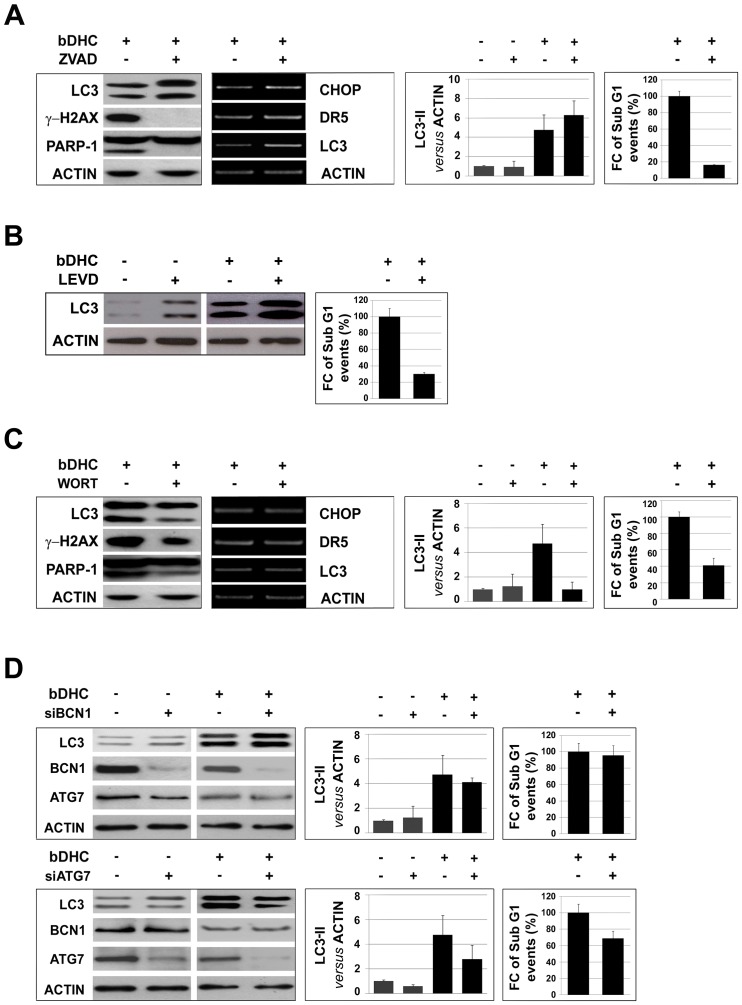
Autophagy activation leads to cell death. **A.** Left panels: LC3, γ-H2AX and PARP1 levels in bDHC cells co-treated with ZVAD. Middle left panel: RT-PCR analysis of the indicated genes normalized *versus* actin mRNA expression. Middle right panel: LC3-II levels *versus* actin with or without co-treatment. Right panels: Flow cytometric analysis of SubG1 events in cells with or without co-treatments. Data are reported as fold change (FC) of SubG1 population relative to bDHC-treated cells (arbitrarily set at 100%). **B.** Left panel: Western blot analysis of LC3 and actin levels following 24 hours co-treatment of DMSO or bDHC with LEVD. Right panel: Fold change (FC) of SubG1 events in cells co-treated with LEVD and bDHC *versus* bDHC-treated cells (arbitrarily set at 100%). **C.** Left panels: LC3, γ-H2AX and PARP1 levels in bDHC cells co-treated with Wortmannin (WORT). Middle left panel: mRNA levels of the indicated genes normalized *versus* actin. Middle right panel: Expression levels of LC3-II normalized to actin with or without co-treatment. Right panels: Flow cytometric analysis of SubG1 events in cells with or without Wortmannin. Data are reported as fold change (FC) of SubG1 population relative to bDHC-treated cells (arbitrarily set at 100%). **D.** Left panels: Western blot analysis of LC3, Beclin1 (BCN1) and Atg7 expression levels in DMSO and bDHC treated cells following BCN1 (upper panel) and Atg7 (lower panel) knock down. Middle panels: LC3-II levels in control, BCN1 and Atg7 inactivated cells have been normalized *versus* actin levels. Values are mean of three independent experiments −/+ SD. Right panels: Fold change (FC) of SubG1 events of BCN1 and Atg7 inactivated bDHC-cells *versus* bDHC-treated cells (arbitrarily set at 100%).

Inhibition of apoptosis by ZVAD resulted in a conspicuous decrease of SubG1 events and of the apoptotic markers, cleaved-PARP-1 and γ-H2AX (Fig. 7A and Fig. S2D, left panel). Compared to bDHC, ZVAD co-treatment didn't reduce the transcriptional activation of CHOP and DR-5, as well as LC3-II protein levels, indicating that ER stress response and the autophagic process are still active when apoptosis is suppressed (Fig. 7A, middle panels, and Fig. S3B, left panel).

The same analysis was performed following co-treatment of bDHC with the specific caspase 4 inhibitor LEVD (Fig. 7B). As well as observed in ZVAD co-treated cells, no decrease of LC3-II levels was detected, while SubG1 events were significantly reduced by LEVD, hinting at an important role of ER stress-related caspase 4 as initiator of bDHC-induced apoptosis.

Pharmacological inhibition of autophagy by co-treatment with Wortmannin, which blocks the early steps of autophagic degradation, resulted in decreased LC3-II levels (Fig. 7C and Fig. S3B, right panel). Concomitantly, we observed a reduction of PARP-1 cleavage, γ-H2AX expression and SubG1 events, but no effects on ER stress genes activated by bDHC (Fig. 7C and Fig. S2D, right panel). Similarly, co-treatment with Chloroquine reduced SubG1 events of about 70% compared to bDHC-treated cells (Fig. S4C).

To further confirm the effects of autophagy inhibition on bDHC-induced cell death, we specifically knocked down the autophagy-related proteins Beclin1 and ATG7 (Fig. 7D). Western blot analysis showed an efficient silencing of both Beclin1 and ATG7, whose expression levels were reduced to about 10% with respect to control cells (Fig. S2E). Interestingly, while Beclin1 inactivation didn't affect either LC3-II levels or apoptosis upon bDHC administration, ATG7 knock down reduced the formation of LC3-II and decreased SubG1 events of bDHC-cells of about 30% (Fig. 7D and Fig. S3C).

These data suggest a role of Beclin1-independent autophagy in bDHC pro-death activity in HCT116 cells.

**Figure 8:**
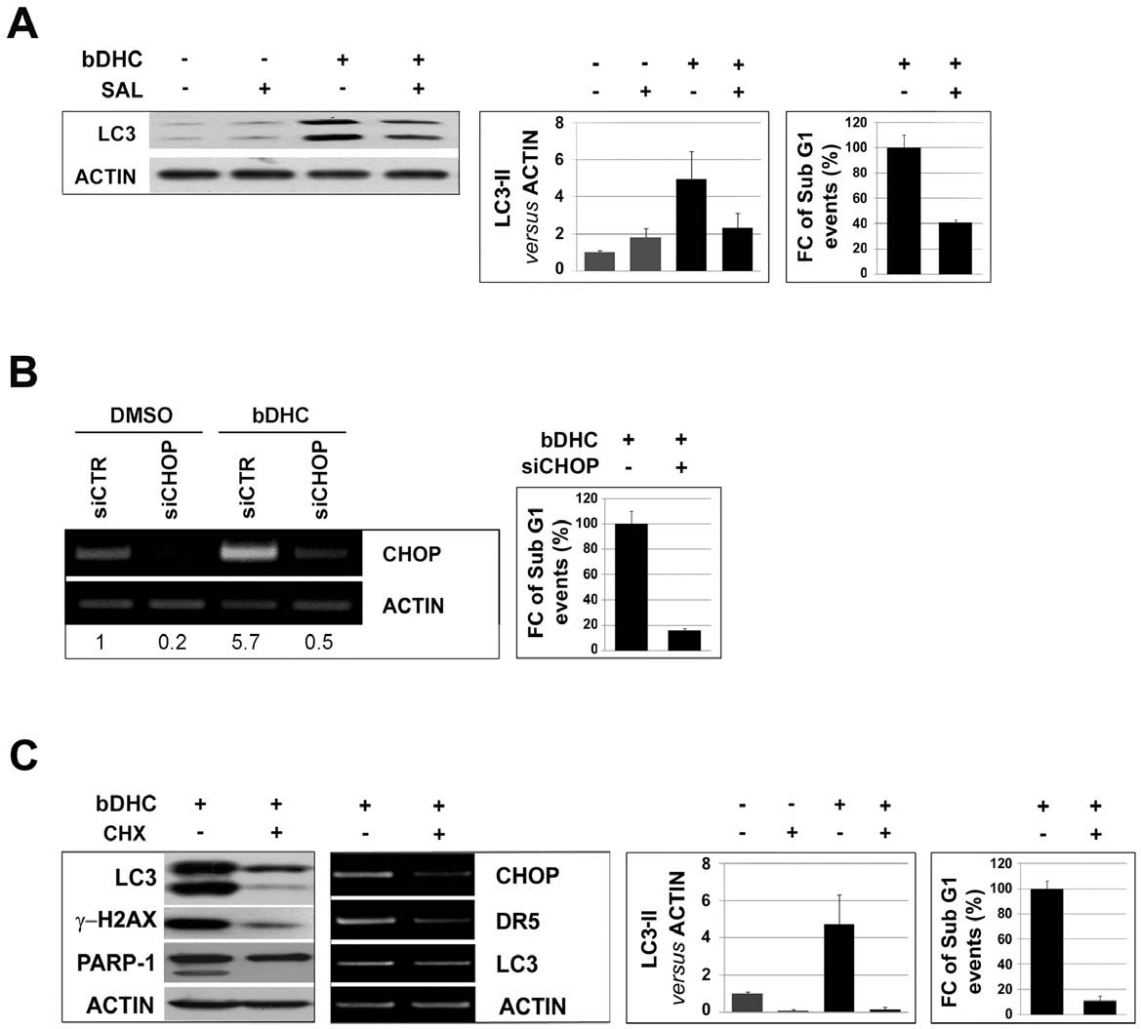
ER stress precedes autophagy-mediated cell death. **A.** Effects of Salubrinal (SAL) on bDHC-treated cells. Protein expression levels of LC3 (left panel) and LC3-II quantification *versus* actin levels (middle panel) in DMSO or bDHC cells co-treated with Salubrinal. Right panel: Fold change (FC) of SubG1 events in co-treated *versus* bDHC-treated cells (arbitrarily considered as 100%). Values are mean of three independent experiments −/+ SD. **B.** Left panel: RT-PCR analysis following CHOP inactivation. Values indicate the quantification of CHOP mRNA levels relative to actin. Right panel: Fold change (FC) of SubG1 events of CHOP inactivated bDHC-cells *versus* bDHC-treated cells (arbitrarily set at 100%). **C.** Effects of co-incubation of bDHC-treated cells with Cycloheximide (CHX). Left and middle left panels: Protein and mRNA expression levels of the indicated genes. Middle right panel: LC3-II levels were quantitated *versus* actin, before and after Cycloheximide co-administration. Right panel: Fold change (FC) of SubG1 events in co-treated *versus* bDHC-treated cells (arbitrarily considered as 100%). Values are mean of three independent experiments −/+ SD.

As shown in Figure 5, bDHC is able to activate ER stress response and accumulation of poly-ubiquitinated proteins. To investigate whether ER stress activation occurred upstream of bDHC-induced autophagy and apoptosis, we used Salubrinal, an ER stress inhibitor, which can regulate eIF2α phosphorylation [65]. Salubrinal co-treatment with bDHC reduced LC3-II levels and SubG1 events (Fig. 8A). CHOP knock down by RNAi reduced apoptosis as well, suggesting the contribution of ER stress to the apoptotic process (Fig. 8B).

Inhibition of translation through Cycloheximide (CHX) co-treatment reduced ER stress activation, as indicated by the lack of overexpression of ER-stress related genes. CHX concurrently lowered the accumulation of LC3-II, as well as γ-H2AX, cleaved-PARP1 and SubG1 events (Fig. 8C, Fig. S2F and Fig. S3D).

Only the co-administration of CHX to cells completely inhibited the accumulation of poly-ubiquitinated proteins induced by bDHC, suggesting that the increase of unfolded nascent proteins can be one of the main causes triggering ER stress response and autophagic-cell death (Fig. S4D).

Taken together, these results indicate that bDHC-induced ER stress leads to autophagy, which occurs upstream of apoptosis and is not protective for bDHC-treated cells.

## Discussion

Apoptosis is the major source to oppose uncontrolled growth of cancer cells and to reduce tumor cell population expansion. Although many dietary agents show interesting chemotherapeutic activity, they often produce major side effects. Curcumin has been used through the ages as alternative medicinal agent and it has been classified as safe by health authorities. It is currently in phase II/III clinical trials, although it shows limited application because of its instability in physiological conditions.

In this study, we demonstrated that the stable Curcumin-derivative bDHC is highly cytotoxic towards colon-cancer cells in a dose-dependent manner. At the concentration of 30 µM, bDHC reduces cell proliferation both by inducing a cell cycle block and by activating the apoptotic program (Fig. 1). At the same concentration, Curcumin is not able to induce such a high cell death in HCT116 cells, but only a G2/M arrest can be detected [20]. It could be that the mismatch repair system protects HCT116 cells from Curcumin cytotoxicity, in part by activating the G2/M checkpoint, as recently shown [66].

bDHC exhibits a selective activity towards cancer cells: in human normal cells (HF and WRL68), it reversibly inhibits proliferation, without eliciting a cytotoxic response (Fig. 2). Its tumor-selectivity can be ascribed to lower uptake in normal cells compared to colon cancer cells (Fig. 2C), but we can't rule out that other mechanisms are involved. As hypothesized for Curcumin, it could be that the great efficacy of bDHC in tumor cells is caused by (i) their lower glutathione levels, which enhances drug sensitivity compared to normal cells or (ii) the constitutive expression of active NF-kB in cancer cells, which is one of Curcuminoids targets [67].

Although it remains to be determined why bDHC is particularly active against colorectal carcinoma cells (HCT116 and LOVO cells) rather than other types of cancer cells [36], [37, VB and CI unpublished data], this is an encouraging prerequisite for bDHC anti-tumor activity *in vivo*. Indeed, Curcumin and its derivatives show increased bioavailability in the gastrointestinal tract and, consequently, slow the growth of gastrointestinal cancers [68], [69].

Our study demonstrates that bDHC-induced apoptosis in colon cancer cells occurs through a mitochondria-dependent pathway (Fig. 3). Mitochondria play an essential role as sensors and amplifiers of death signaling pathways. In particular, the collapse of the mitochondrial membrane potential is considered an irreversible point in the death cascade [70]. Following bDHC treatment, we observed a decrease of mitochondrial transmembrane potential (Fig. 3), and the release into the cytosol of Cytochrome C, which triggers the caspase-activating cell death pathways [71] (Fig. 3C). Cytochrome C is a key player of mitochondria-dependent apoptotic cell death: its release from mitochondria elicits the formation of Apaf-1/caspase 9 apoptosome, which further activates effector caspases 3 and 7, leading to the cleavage of nuclear substrates, such as PARP-1 (Fig. 3B) and lamins, and to oligonucleosomal DNA fragmentation [72].

We showed a major role of caspases as final effectors of bDHC-cell death (Fig. 3B). The inhibition of pro-caspases cleavage by ZVAD completely suppresses bDHC-induced apoptosis, the cleavage of PARP-1 and the phosphorylation of H2AX at Ser139 (Fig. 3A-B and Fig. 7A). Even if the function of γH2AX has been mainly linked to DNA-damage repair [73], this histone plays a key role in programmed cell death. Its formation during apoptosis depends on caspases activation and is concurrent with the initiation of apoptotic endonuclease activation [45], regulating the accessibility of various DNases to DNA [74], [75].

The balance between cell proliferation and cell death is controlled by anti- and pro-apoptotic proteins, most of which are known p53 target genes. RT-PCR analysis indicates that bDHC can induce cell death by regulating the expression of the Bcl-2 family members: Bcl-2α and Bcl-XL anti-apoptotic proteins are down-regulated, while the pro-apoptotic Noxa and Puma genes are up-regulated by bDHC (Fig. 4A).

Although the pro-apoptotic Bax doesn't show any transcriptional activation, bDHC treatment of HCT116 Bax−/− cells clearly highlights its contribution to the activation of cell death (Fig. 4A and C). Indeed, Bax-induced mitochondrial membrane permeabilization and the resulting Cytochrome C release into the cytoplasm, can be achieved through Bax translocation from the cytoplasm to the outer mitochondrial membrane, without any p53-transcriptional activation [76]. The analysis of p53 expression and cellular compartmentalization shows that protein levels increase exclusively into the cytoplasm, suggesting a marginal role for p53 in the transcriptional regulation of anti- and pro-apoptotic target genes in response to bDHC (Fig. 4D and E). A p53 transcriptional-independent activation of Bax has been described following the activation of caspase 8 and the up-regulation of Puma [77], consistently with our data from bDHC-treated cells (Fig. 3 and Fig. 4). In addition to Bax, also Puma and Noxa activation can be achieved through p53-independent mechanisms: Puma was shown to be induced by ER stress [49] and Noxa by proteasome inhibition [78], both injuries being observed upon bDHC administration to HCT116 cells (Fig. 5). The increase of p53 expression upon apoptosis inhibition by ZVAD, which results in a G2/M cell cycle arrest, ascribes to the oncosuppressor mainly a role in bDHC-cell cycle block rather than apoptosis (Fig. 3A). The same behavior is observed for p21, which has a fundamental function in maintaining the cell cycle block induced by Curcumin and its derivatives [20].

Administration of bDHC to HCT116/E6 cells partially reduces but doesn't abolish apoptosis, suggesting that a p53-independent-cell death can occur. On the other hand, p53 has come to light as a positive regulator of autophagy, which indeed is not induced by bDHC in HCT116/E6 (Fig. S4B). We believe that the halved percentage of SubG1 population in E6 compared to wt cells could be ascribed to the lack of autophagy activation, which contributes to bDHC-cell death.

RT-PCRs highlight the activation of various genes involved in ER stress response: CHOP, ATF6, GRP78, HERPUD1 and sXBP1 mRNA levels increase already after 8 hours of bDHC treatment (Fig. 5). The activation of ER stress response, and in particular of CHOP, could determine the transcriptional down-regulation of the anti-apoptotic Bcl-2, in a p53-independent manner.

Murine caspase 12 and human caspase 4 have been described to be cleaved by ER stress inducing agents, and to participate to ER-induced apoptosis [55], [56]. Moreover, human cells treated with siRNA targeting caspase 4 were resistant to ER-stress induced apoptosis [55].

In agreement with these data, we detected the cleavage of caspase 4 following bDHC administration to HCT116 cells, and a clear decrease of apoptosis was determined by preventing its activation with LEVD (Fig. 3C and Fig. 7B). Consistently with recent results demonstrating a novel ER stress-triggered caspase cascade initiated by caspase 4 and involving caspase 8 [79], LEVD co-administration to cells resulted in a decrease not only of cleaved-caspase 4, but also of cleaved-caspase 8, suggesting that ER stress-induced caspase 4 leads to caspase 8 processing in HCT116 cells (Fig. 3C).

Accumulating data indicate that ER stress can trigger autophagy [80], [81], [82]. In the case of Unfolded Protein Response (UPR), stimulation of autophagy can be required to activate the cell death machinery [83]. The UPR and autophagic process can work independently from each other, or they can share their cytoprotective or cytocidial functions, depending on the type and duration of the cellular stress [84].

The intricate cross-talk between apoptosis and autophagy is crucial to the overall fate of the cell. Indeed, the final outcome of autophagy depends on (i) the stress-inducing stimulus and (ii) the cellular context: autophagy can help ER-stressed cells to survive, contributing to the elimination of unfolded proteins, or can take part to ER stress induced cell death [85].

In this study we have shown that autophagy induced by bDHC is a consequence of ER stress and has an important role in the activation of cell death. In fact, when apoptosis is inhibited through ZVAD and LEVD, ER stress and autophagy are still active in bDHC-treated cells. On the other hand, following pharmacological inhibition of autophagy by Wortmannin or Chloroquine, apoptosis is strongly attenuated, suggesting that bDHC-induced autophagy occurs upstream of apoptosis (Fig. 7C and Fig. S4C). To better investigate the role of autophagy, we inactivated both Beclin1 and ATG7, known autophagy related genes. The analysis of LC3-II levels upon RNAi in bDHC cells highlights that Beclin1 has a marginal function in bDHC-autophagy, and consequently its inactivation doesn't prevent autophagic cell death. This was quite expected considering that Beclin1 has been mainly involved in starvation- rather than drug-induced autophagy. Moreover, decreased levels of Beclin1 protein observed following bDHC administration are consistent with its exclusion from bDHC-autophagy process (Fig. 7D). In particular, time-course analysis shows that Beclin1 levels are already reduced upon 8 hours of bDHC treatment, before autophagy is activated (data not shown). In contrast to Beclin1, Atg7 silencing results in a significant decrease in LC3-II accumulation and alters cell death response to bDHC.

Although the suppression of autophagy reduces SubG1 population, neither Wortmannin/Chloroquine nor ATG7 knockdown can completely rescue the loss of cell viability caused by bDHC, hinting at an independent apoptotic pathway running parallel to the autophagic-mediated cell death. This is also consistent with the percentage of apoptosis detected in HCT116/E6, which can't activate autophagy (Fig. S4B). Nevertheless, both autophagic-dependent and -independent cell death seems to be triggered by ER stress.

The increase of poly-ubiquitinated proteins in bDHC treated cells, fully suppressed by Cycloheximide (Fig. S4D), hints at a possible activity of bDHC as proteasome inhibitor, as well as reported for Curcumin [59], [60]. The lack of p53 doesn't reduce proteins poly-ubiquitination, suggesting once again that p53 has a possible role downstream ER stress response in the activation of autophagic-cell death.

Although bDHC has shown enhanced anti-proliferative activity compared to Curcumin in HCT116 and LOVO cells, its high IC50 concentration could limit its possible development in clinical application. With the purpose to reduce the dose necessary to activate bDHC-cell death in tumor cells, we have recently designed and tested a bDHC-analog, by inserting an alkali group in C-3 position of bDHC backbone (C3-bDHC) [86]. C3-bDHC has improved chemical stability in physiological conditions and it exerts anti-proliferative activity in HCT116 and LOVO cells at 10-fold lower concentration compared to bDHC (IC50 = 3 µM in HCT116 and 4 µM in LOVO cells) [86]. Our preliminary data support that C3-bDHC is able to induce autophagy and cell death in HCT116 even better than its lead compound (Fig. S5).

Overall, we demonstrated that bDHC exerts a selective cytotoxic activity on colon cancer cells, through the activation of ER-stress induced autophagic cell death, providing new evidence that autophagy is an effective inducer of cell death.

Further studies are needed to better characterize bDHC and its derivatives [86], [87], new possible candidates for promising therapies to prevent or treat colon cancer diseases through their pro-autophagic activity.

## Supporting Information

Figure S1 **A.** Percentages of 4n phospho-Ser10H3 positive HCT116 cells (black bars) counted out of a total number of 4n cells (grey bars) after DMSO, Curcumin and bDHC treatments for 16 and 24 hours. **B.** Scanning Electron Microscopy images of control and bDHC-treated HCT116 cells. Scale bar: 5 µm. **C.** Left panel: Distribution of LOVO cells into the different phases of the cell cycle following treatment with DMSO or bDHC for 24 hours. The percentages are means of three independent experiments −/+ SD. Right panel: γH2AX expression levels in LOVO cells treated with bDHC compared to DMSO control cells. Actin was used as loading control. **D.** Western blot analysis of γH2AX expression following bDHC administration to HF (left panel) and WRL68 cells (right panel) for 24 and 48 hours. Actin was used as loading control.
(TIF)

Figure S2 **A.** Quantification of the expression levels of γH2AX, p53 and p21 *versus* actin in HCT116 cells co-treated with ZVAD or incubated with DMSO and bDHC alone (Western blots shown in Fig. 3A). **B.** Cytoplasmic (left panel) and mitochondrial/nuclear Cytochrome C levels following time-dependent administration of bDHC. Adriamycin (ADR) was used as positive control (Western blots shown in Fig. 3C). **C.** Quantification of the expression levels of Bcl2α, Bcl-XL and BAX *versus* actin in total cellular extracts of DMSO and bDHC treated cells (Western blots shown in Fig. 4A). **D.** Expression levels of γH2AX and cleaved PARP-1 in HCT116 cells co-treated with ZVAD (left panel) or Wortmannin (WORT) and bDHC (Western blot shown in Fig. 7A). **E.** Beclin1 (BCN1) and ATG7 expression levels normalized to actin levels in BCN1 (left panel) and ATG7 (right panel) silenced cells untreated or treated with bDHC (Western blots shown in Fig. 7B). **F.** Quantification of the expression levels of γH2AX and cleaved PARP1 *versus* actin in HCT116 cells treated with bDHC or co-incubated with Cycloheximide (CHX) and bDHC (Western blots shown in Fig. 8C). All the indicated values are mean of at least three independent experiments −/+ SD.
(TIF)

Figure S3 **A.** Ratio of LC3-II/LC3-I expression levels normalized to actin following DMSO and time dependent exposure of HCT116 cells to bDHC. **B.** LC3-II/LC3-I ratio in DMSO and bDHC cells co-treated with ZVAD or Wortmannin (WORT) *versus* DMSO. **C.** LC3-II/LC3-I ratio in Beclin1 (BCN1) and ATG7 inactivated cells following DMSO or bDHC administration. **D.** Ratio of LC3-II/LC3-I expression levels upon co-incubation of Cycloheximide (CHX) with bDHC compared to DMSO. Basal LC3-II/LC3-I ratio in control cells has been arbitrarily set at 1. All the indicated values are means of three independent experiments, *P<0.05, **P<0.01.
(TIF)

Figure S4 **A.** Left panel: Western blot analysis of LC3 expression following 24 and 48 hours of bDHC exposure in HF cells. Right panel indicates the levels of LC3-II *versus* actin following time-dependent exposure to bDHC. Values are means of three independent experiments. **B.** Upper left panels: LC3 expression levels in HCT116/E6 compared to HCT116 cells. Upper right panel: Quantification of LC3-II *versus* actin levels in HCT116/E6 cells incubated with bDHC for 16 and 24 hours. Lower left panel: Distribution of HCT116/E6 cells throughout the different phases of the cell cycle (PI monoparametric analysis), following DMSO and bDHC administration for 24 hours. Lower right panel: Western blot analysis of poly-ubiquitinated proteins in HCT116/E6 after bDHC treatment for 24 hours. **C.** Left panel: LC3, γ-H2AX and PARP1 levels in DMSO and bDHC cells co-treated with Chloroquine (CQ). Actin was used as loading control. Middle panel: LC3-II/LC3-I ratio in DMSO and bDHC cells co-treated with Chloroquine. LC3-II/LC3-I ratio in DMSO cells has been arbitrarily set at 1. Values are means of three independent experiments, *P<0.05, **P<0.01. Right panel: Fold change (FC) of SubG1 events of HCT116 cells co-incubated with bDHC and Chloroquine (CQ) compared to bDHC-treated cells (arbitrarily considered as 100%). **D.** Left panel: Western blot with anti-ubiquitin antibody of total cellular extracts of DMSO and bDHC-treated cells co-incubated with ZVAD, Wortmannin, Chloroquine and Cycloheximide. Middle panel: Time-dependent effect of Salubrinal co-treatment on proteins poly-ubiquitination. Actin was used as internal loading control. Right panel: Western blot of poly-ubiquitinated proteins in HCT116 total extracts after CHOP inactivation, with or without bDHC co-treatment.
(TIF)

Figure S5 Left panel: Western blot analysis of LC3 expression in HCT116 cells treated with DMSO, bDHC and C3-bDHC for 24 hours. Right panel: PI/FACS cell cycle analysis of HCT116 cells following incubation with DMSO and C3-bDHC for 24 hours. Values are means of three independent experiments −/+ SD.
(TIF)
