# Supplementary figures and images for: bis-Dehydroxy-Curcumin Triggers Mitochondrial-Associated Cell Death in Human Colon Cancer Cells through ER-Stress Induced Autophagy
Source: PLoS One. 2013 Jan 11;8(1):e53664. doi: 10.1371/journal.pone.0053664 (PMC3543386; doi:10.1371/journal.pone.0053664)

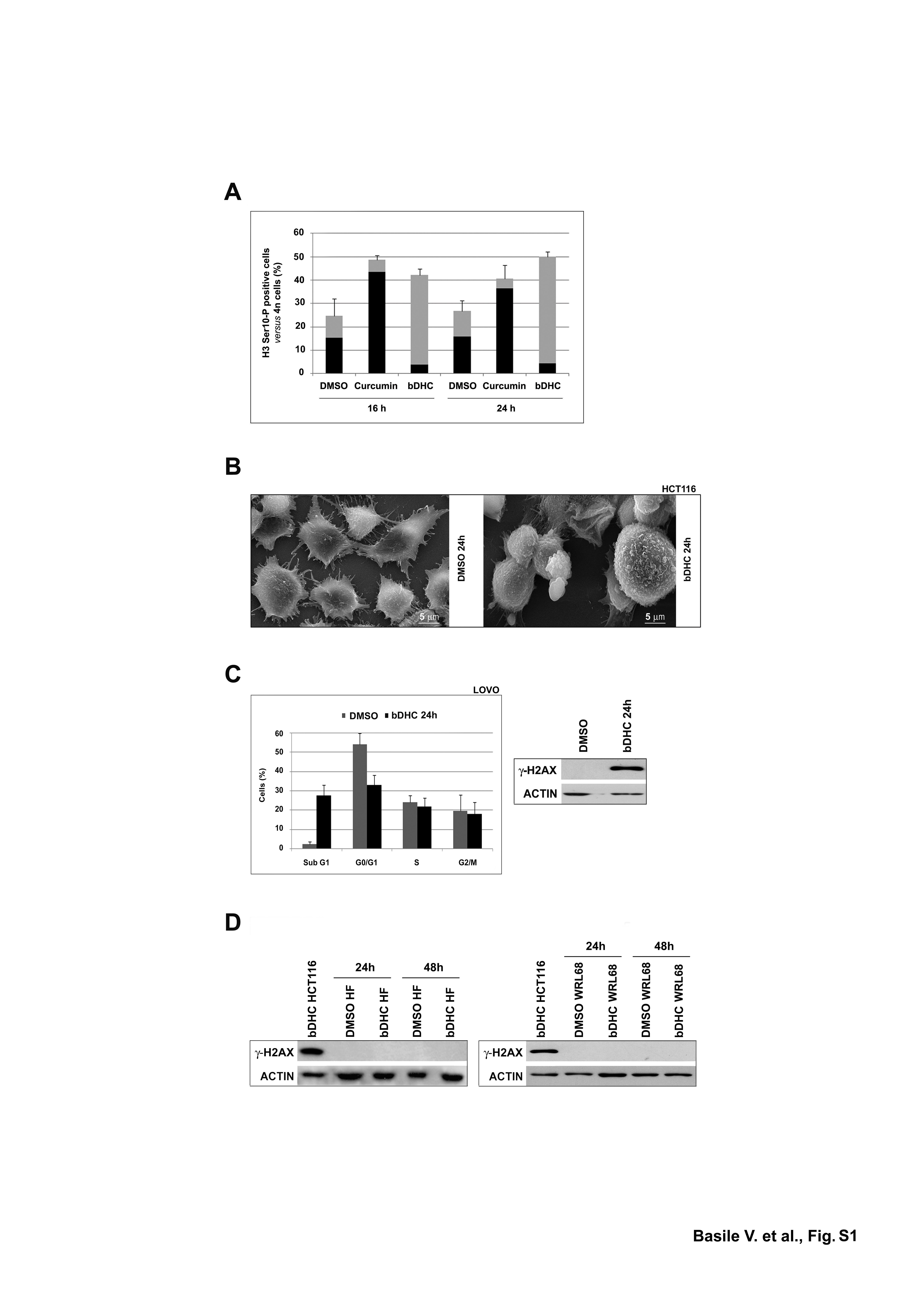

Supplement: Figure S1 — A. Percentages of 4n phospho-Ser10H3 positive HCT116 cells (black bars) counted out of a total number of 4n cells (grey bars) after DMSO, Curcumin and bDHC treatments for 16 and 24 hours. B. Scanning Electron Microscopy images of control and bDHC-treated HCT116 cells. Scale bar: 5 µm. C. Left panel: Distribution of LOVO cells into the different phases of the cell cycle following treatment with DMSO or bDHC for 24 hours. The percentages are means of three independent experiments −/+ SD. Right panel: γH2AX expression levels in LOVO cells treated with bDHC compared to DMSO control cells. Actin was used as loading control. D. Western blot analysis of γH2AX expression following bDHC administration to HF (left panel) and WRL68 cells (right panel) for 24 and 48 hours. Actin was used as loading control. (TIF) [file pone.0053664.s001.tif]

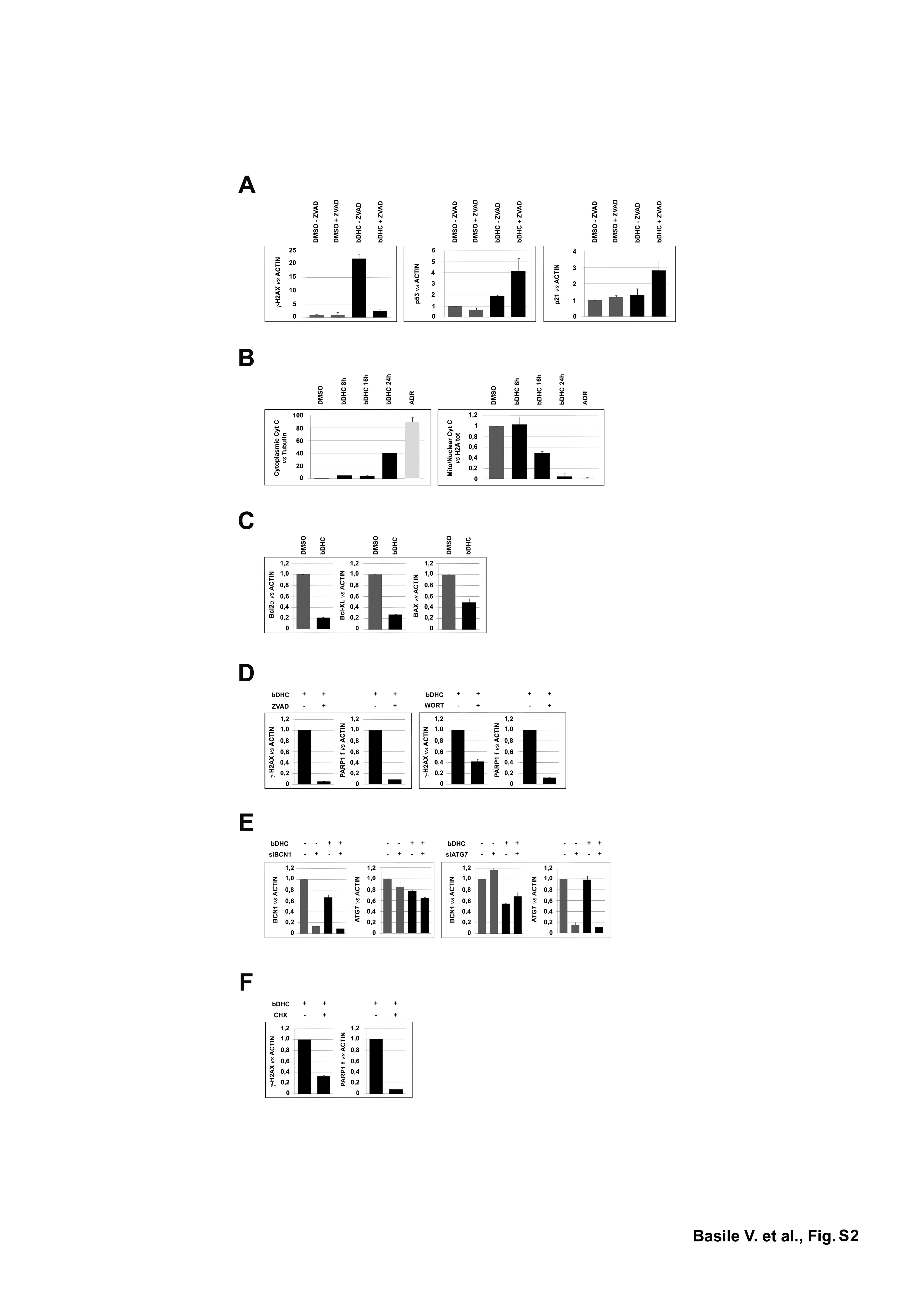

Supplement: Figure S2 — A. Quantification of the expression levels of γH2AX, p53 and p21 versus actin in HCT116 cells co-treated with ZVAD or incubated with DMSO and bDHC alone (Western blots shown in Fig. 3A). B. Cytoplasmic (left panel) and mitochondrial/nuclear Cytochrome C levels following time-dependent administration of bDHC. Adriamycin (ADR) was used as positive control (Western blots shown in Fig. 3C). C. Quantification of the expression levels of Bcl2α, Bcl-XL and BAX versus actin in total cellular extracts of DMSO and bDHC treated cells (Western blots shown in Fig. 4A). D. Expression levels of γH2AX and cleaved PARP-1 in HCT116 cells co-treated with ZVAD (left panel) or Wortmannin (WORT) and bDHC (Western blot shown in Fig. 7A). E. Beclin1 (BCN1) and ATG7 expression levels normalized to actin levels in BCN1 (left panel) and ATG7 (right panel) silenced cells untreated or treated with bDHC (Western blots shown in Fig. 7B). F. Quantification of the expression levels of γH2AX and cleaved PARP1 versus actin in HCT116 cells treated with bDHC or co-incubated with Cycloheximide (CHX) and bDHC (Western blots shown in Fig. 8C). All the indicated values are mean of at least three independent experiments −/+ SD. (TIF) [file pone.0053664.s002.tif]

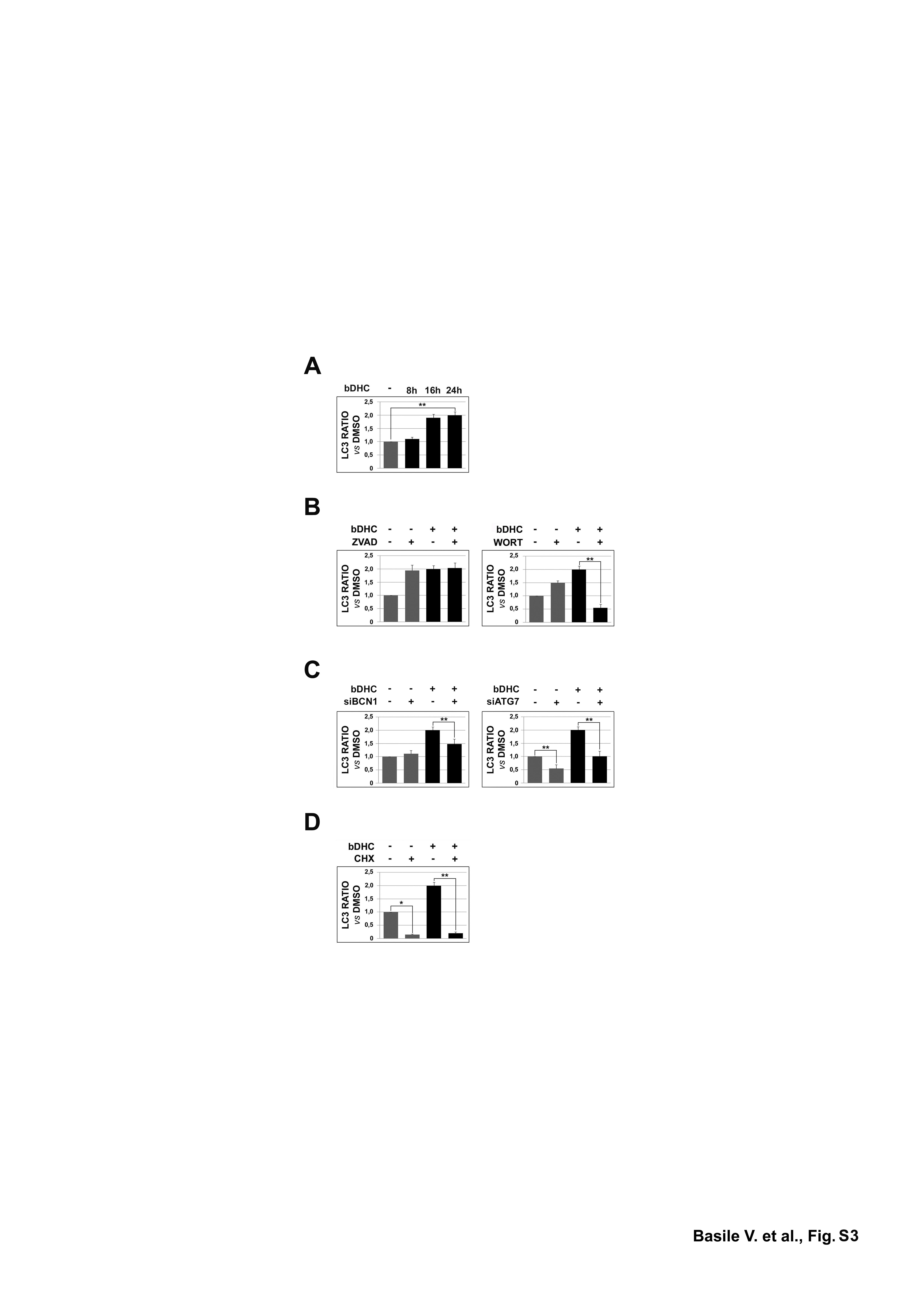

Supplement: Figure S3 — A. Ratio of LC3-II/LC3-I expression levels normalized to actin following DMSO and time dependent exposure of HCT116 cells to bDHC. B. LC3-II/LC3-I ratio in DMSO and bDHC cells co-treated with ZVAD or Wortmannin (WORT) versus DMSO. C. LC3-II/LC3-I ratio in Beclin1 (BCN1) and ATG7 inactivated cells following DMSO or bDHC administration. D. Ratio of LC3-II/LC3-I expression levels upon co-incubation of Cycloheximide (CHX) with bDHC compared to DMSO. Basal LC3-II/LC3-I ratio in control cells has been arbitrarily set at 1. All the indicated values are means of three independent experiments, *P<0.05, **P<0.01. (TIF) [file pone.0053664.s003.tif]

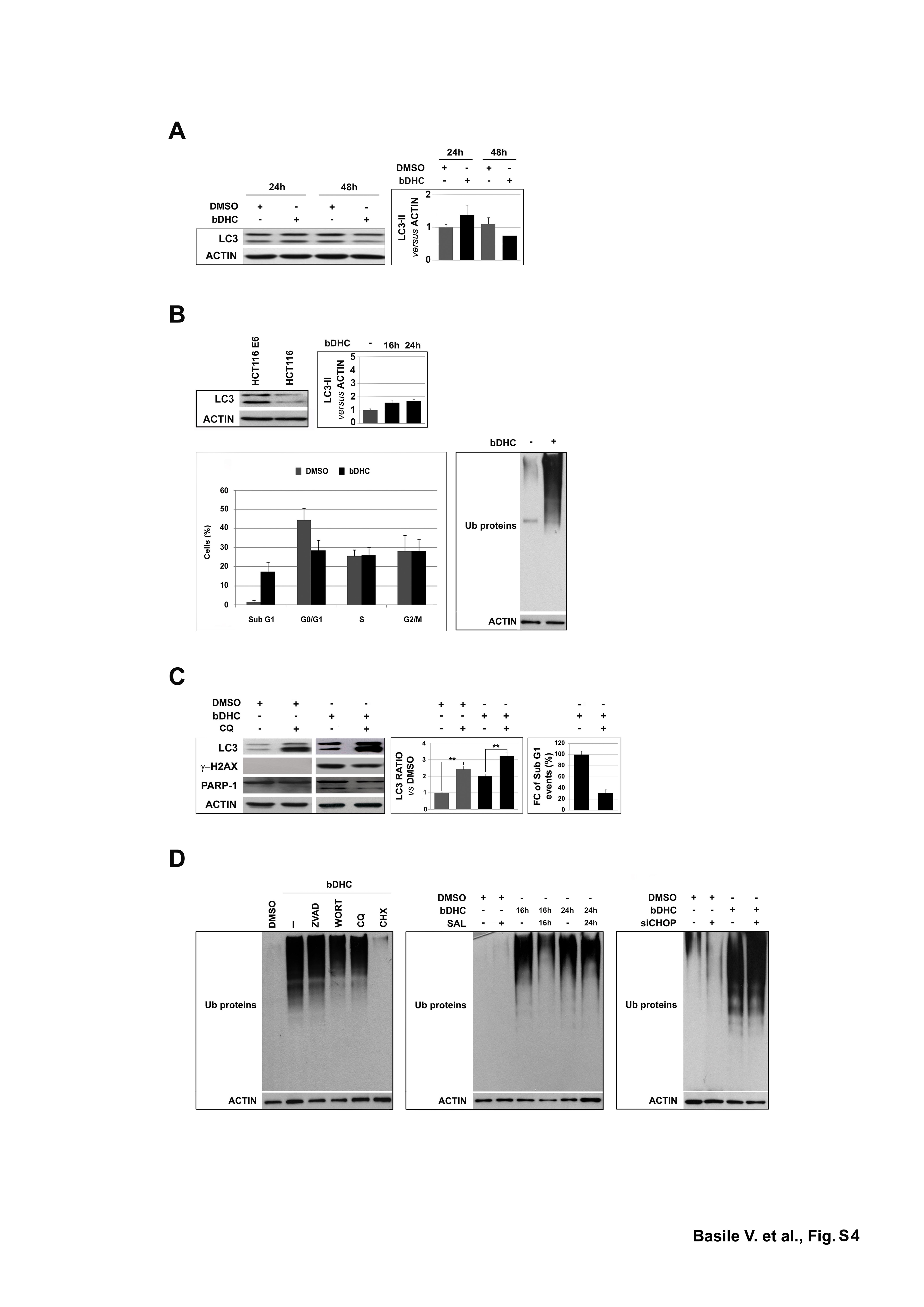

Supplement: Figure S4 — A. Left panel: Western blot analysis of LC3 expression following 24 and 48 hours of bDHC exposure in HF cells. Right panel indicates the levels of LC3-II versus actin following time-dependent exposure to bDHC. Values are means of three independent experiments. B. Upper left panels: LC3 expression levels in HCT116/E6 compared to HCT116 cells. Upper right panel: Quantification of LC3-II versus actin levels in HCT116/E6 cells incubated with bDHC for 16 and 24 hours. Lower left panel: Distribution of HCT116/E6 cells throughout the different phases of the cell cycle (PI monoparametric analysis), following DMSO and bDHC administration for 24 hours. Lower right panel: Western blot analysis of poly-ubiquitinated proteins in HCT116/E6 after bDHC treatment for 24 hours. C. Left panel: LC3, γ-H2AX and PARP1 levels in DMSO and bDHC cells co-treated with Chloroquine (CQ). Actin was used as loading control. Middle panel: LC3-II/LC3-I ratio in DMSO and bDHC cells co-treated with Chloroquine. LC3-II/LC3-I ratio in DMSO cells has been arbitrarily set at 1. Values are means of three independent experiments, *P<0.05, **P<0.01. Right panel: Fold change (FC) of SubG1 events of HCT116 cells co-incubated with bDHC and Chloroquine (CQ) compared to bDHC-treated cells (arbitrarily considered as 100%). D. Left panel: Western blot with anti-ubiquitin antibody of total cellular extracts of DMSO and bDHC-treated cells co-incubated with ZVAD, Wortmannin, Chloroquine and Cycloheximide. Middle panel: Time-dependent effect of Salubrinal co-treatment on proteins poly-ubiquitination. Actin was used as internal loading control. Right panel: Western blot of poly-ubiquitinated proteins in HCT116 total extracts after CHOP inactivation, with or without bDHC co-treatment. (TIF) [file pone.0053664.s004.tif]

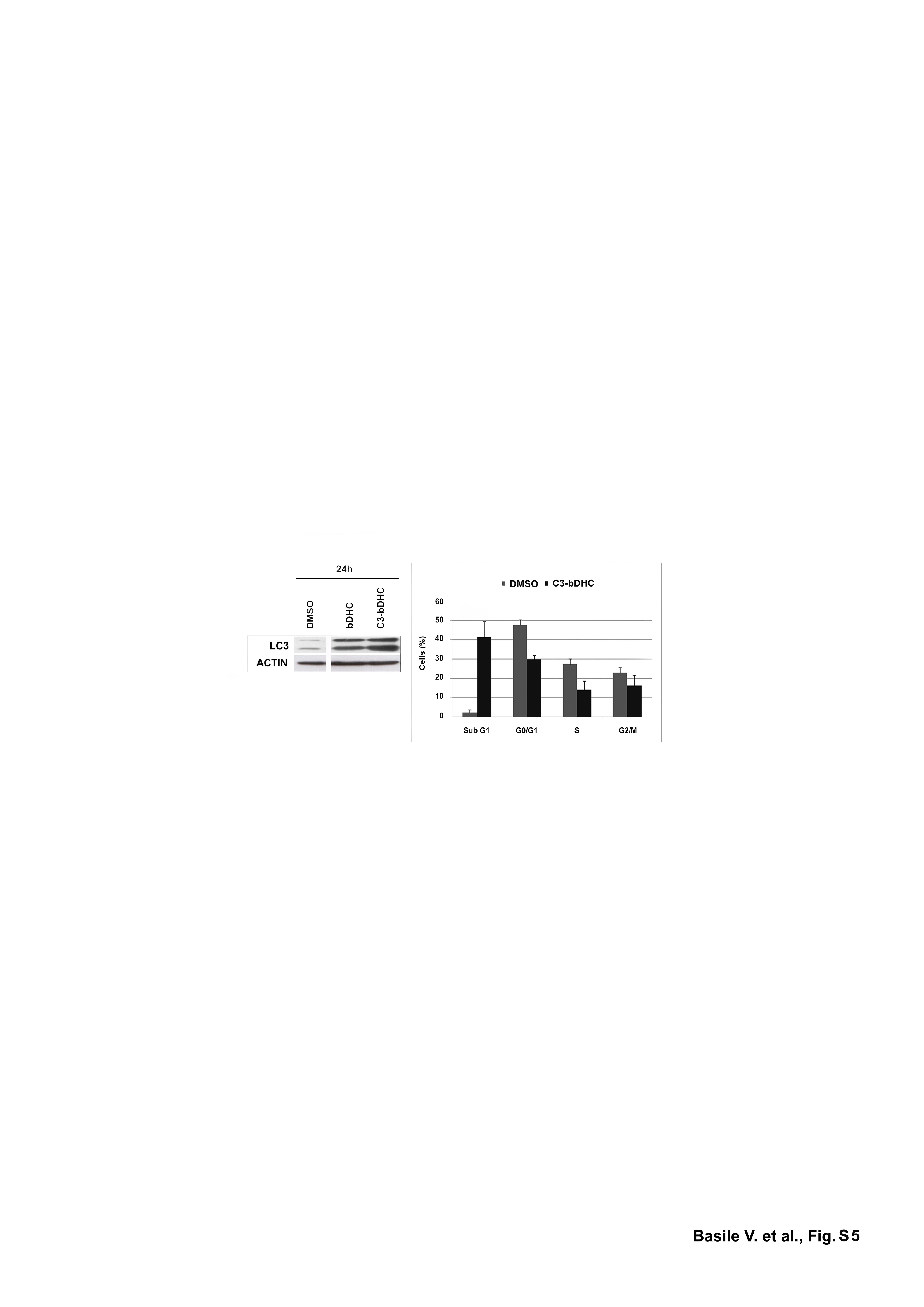

Supplement: Figure S5 — Left panel: Western blot analysis of LC3 expression in HCT116 cells treated with DMSO, bDHC and C3-bDHC for 24 hours. Right panel: PI/FACS cell cycle analysis of HCT116 cells following incubation with DMSO and C3-bDHC for 24 hours. Values are means of three independent experiments −/+ SD. (TIF) [file pone.0053664.s005.tif]
